# Common Transcriptional Mechanisms for Visual Photoreceptor Cell Differentiation among Pancrustaceans

**DOI:** 10.1371/journal.pgen.1004484

**Published:** 2014-07-03

**Authors:** Simpla Mahato, Shinichi Morita, Abraham E. Tucker, Xulong Liang, Magdalena Jackowska, Markus Friedrich, Yasuhiro Shiga, Andrew C. Zelhof

**Affiliations:** 1 Department of Biology, Indiana University, Bloomington, Indiana, United States of America; 2 School of Life Sciences, Tokyo University of Pharmacy and Life Sciences, Hachioji, Tokyo, Japan; 3 Department of Biology, Southern Arkansas University, Magnolia, Arkansas, United States of America; 4 Department of Biological Sciences, Wayne State University, Detroit, Michigan, United States of America; 5 Department of Anatomy and Cell Biology, Wayne State University, School of Medicine, Detroit, Michigan, United States of America; New York University, United States of America

## Abstract

A hallmark of visual rhabdomeric photoreceptors is the expression of a rhabdomeric opsin and uniquely associated phototransduction molecules, which are incorporated into a specialized expanded apical membrane, the rhabdomere. Given the extensive utilization of rhabdomeric photoreceptors in the eyes of protostomes, here we address whether a common transcriptional mechanism exists for the differentiation of rhabdomeric photoreceptors. In *Drosophila*, the transcription factors Pph13 and Orthodenticle (Otd) direct both aspects of differentiation: rhabdomeric opsin transcription and rhabdomere morphogenesis. We demonstrate that the orthologs of both proteins are expressed in the visual systems of the distantly related arthropod species *Tribolium castaneum* and *Daphnia magna* and that their functional roles are similar in these species. In particular, we establish that the Pph13 homologs have the ability to bind a subset of Rhodopsin core sequence I sites and that these sites are present in key phototransduction genes of both *Tribolium* and *Daphnia*. Furthermore, Pph13 and Otd orthologs are capable of executing deeply conserved functions of photoreceptor differentiation as evidenced by the ability to rescue their respective *Drosophila* mutant phenotypes. Pph13 homologs are equivalent in their ability to direct both rhabdomere morphogenesis and opsin expression within *Drosophila*, whereas Otd paralogs demonstrate differential abilities to regulate photoreceptor differentiation. Finally, loss-of-function analyses in *Tribolium* confirm the conserved requirement of Pph13 and Otd in regulating both rhabdomeric opsin transcription and rhabdomere morphogenesis. Taken together, our data identify components of a regulatory framework for rhabdomeric photoreceptor differentiation in Pancrustaceans, providing a foundation for defining ancestral regulatory modules of rhabdomeric photoreceptor differentiation.

## Author Summary

Visual systems are populated by one of two fundamental types of photoreceptors, ciliary and rhabdomeric. Each photoreceptor type is defined by the opsin molecule expressed and the final morphological form adapted to house the phototransduction machinery. Here we address whether a common transcriptional mechanisms exists for the differentiation of rhabdomeric photoreceptors. We demonstrate that orthologs of two *Drosophila* (fruit fly) transcription factors, Pph13 and Orthodenticle, are expressed in photoreceptors of Pancrustaceans, *Tribolium* (red flour beetle) and *Daphnia* (water flea), and are capable of executing conserved functions of rhabdomeric photoreceptor differentiation. In particular, *Tribolium* and *Daphnia* orthologs are capable of substituting and rescuing the photoreceptor differentiation defects observed in their corresponding *Drosophila* mutants. Furthermore, loss of function analysis in *Tribolium* of both *Pph13* and *orthodenticle* genes demonstrate they regulate opsin transcription and morphogenesis of the photoreceptor apical membrane. Our data illuminate a framework for rhabdomeric photoreceptor differentiation and provide the foundation for defining the ancestral regulatory modules for rhabdomeric differentiation and potential modifications that underlie the functional diversity observed in rhabdomeric photoreceptors.

## Introduction

Rhabdomeric (r) photoreceptors are one of two fundamental types of photoreceptors that have been described [1]. Typically, r- photoreceptors populate the visual systems of protostomes including insects, crustaceans, and annelids (reviewed in [2]). This wide phylogenetic distribution and the presence of both types of photoreceptors in many species imply that r- photoreceptors, like their deuterostome counterparts, ciliary photoreceptors, were present before the split of bilaterian animals [3]–[6]. Despite this wide utilization of r- photoreceptors, knowledge about r- photoreceptor differentiation has been virtually exclusively defined from studies in the *Drosophila* (fruit fly) visual system (reviewed in [7], [8]). Generally, two features characterize r- photoreceptor differentiation. The first is the expression of an r- opsin for light detection, which upon the absorption of a photon leads to the activation of a Phospholipase C cascade and the depolarization of the photoreceptor. This phototransduction cascade permits the amplification of responses to single photons of light [9]. The second program concerns the generation of the rhabdomere, an expansion of the photoreceptor apical membrane to house the phototransduction machinery. This adaptation is necessary for increasing the accuracy of measuring light intensity required for vision [10]. Therefore, understanding the development and evolution of rhabdomeric photoreceptor differentiation requires clarification of how these processes are transcriptionally regulated and whether this regulation is conserved within all rhabdomeric photoreceptor types.

In *Drosophila*, two homeodomain proteins have been identified that are critical for regulating r- photoreceptor differentiation. The first, Orthodenticle (Otd), is the *Drosophila* ortholog of a conserved family of Otd/Otx homeodomain transcription factors, which are essential for head and brain development across species [11], [12]. In the r- photoreceptors of *Drosophila* eyes, *otd* is required for both aspects of differentiation [13]. Otd promotes the proper morphogenesis of rhabdomeres and directs multiple aspects of the differential expression of r- opsin paralogs, which characterizes the complex visual organization of the *Drosophila* retinas (for a review see [14]). In particular, Otd is required for the expression of *rh3*, an ultra-violet (UV) sensitive r- opsin, and *rh5*, a blue (B) sensitive r- opsin in the two inner photoreceptors of *Drosophila* ommatidia [15], [16]. In addition, Otd is critical for repressing *rh6*, the *Drosophila* ancestral long-wave (LW) opsin [17] in the six outer photoreceptors of the *Drosophila* ommatidium [16].

The second critical transcription factor is PvuII-PstI homology 13 (Pph13), a paired-class homeobox protein that is similar to the vertebrate Aristaless-related homeodomain (Arx) proteins [18], [19]. Like Otd, the loss of Pph13 results in defects in rhabdomere morphogenesis in the *Drosophila* eye [19]. Interestingly, the concurrent removal of Pph13 and Otd results in complete elimination of the rhabdomeres in *Drosophila*, suggesting that the two proteins cooperate and have overlapping functions with respect to photoreceptor morphology [20]. Pph13 is also essential for the expression of r- opsins *rh6* and *rh2* [20]. In contrast to *otd* mutants, phototransduction is abolished in *Pph13* mutants due to the loss and reduced transcription of several key components of the phototransduction machinery [19], [20]. Lastly, Pph13 regulates photoreceptor differentiation by binding to a subset of Rhodopsin core sequence I (RCSI) elements [20], [21], which are conserved elements present in *Drosophila* Rhodopsin promoters [22]–[24]. Consistent with this, *Drosophila* Pph13 is necessary and sufficient for driving photoreceptor specific reporter gene expression from the artificial 3XP3 promoter [20], which has been assembled from a Pax6 homeodomain binding site [25]. Given the central role of both Pph13 and Otd in r- photoreceptor differentiation in *Drosophila*, the question we address here is whether Pph13 and Otd functions represent a common regulatory pathway of arthropod r-visual photoreceptor differentiation.

To examine whether Pph13 and Otd could represent a common set of transcription factors required for r- visual photoreceptor differentiation, we chose to investigate their orthologs from two key nodal species, *Tribolium castaneum* (red flour beetle), a second insect, and *Daphnia* (water flea), a crustacean. Together, insects and crustaceans define the superclade Pancrustacea within the Arthropoda [26], [27] and any similarities between *Daphnia*, *Tribolium*, and *Drosophila* r- photoreceptor differentiation would indicate a pathway common to the ancestor that generated both lineages, at least 500 million years ago [28], [29]. First, we demonstrate that Otd and Pph13 orthologs are present and expressed in the visual systems of both species. Consistent with conservation of Pph13 mediated r- opsin regulation, the Pph13 RCSI binding site is conserved in the promoters of the r-opsin genes of both *Tribolium* and *Daphnia* and found only within LW r- opsins. Further, the *Tribolium* and *Daphnia* Pph13 homologs have retained similar DNA binding capabilities to their respective endogenous RCSI sites and we confirmed their functional equivalency to direct photoreceptor differentiation in *Drosophila* photoreceptors by transgenic rescue. The Otd paralogs of *Tribolium* and *Daphnia* are comparable in their ability to direct rhabdomere morphogenesis but exhibit differential abilities with respect to r- opsin regulation in *Drosophila*. Lastly, functional analyses in *Tribolium* reveal that both Pph13 and Otd homologs are essential for both aspects of photoreceptor differentiation, rhabdomere creation and r-opsin expression. In particular, Pph13 is a critical factor for *LW* r-opsin expression and Otd2 is necessary for the transcription of *UV* sensitive r-opsin. In summary, our data identify common components for rhabdomeric photoreceptor differentiation among Pancrustaceans, providing a foundation for defining the ancestral transcriptional mechanisms for rhabdomeric photoreceptor differentiation throughout Bilateria.

## Results

### Evolutionary conservation of the *Pph13* and *orthodenticle* transcription factor genes in insects and crustaceans

**Figure 1:**
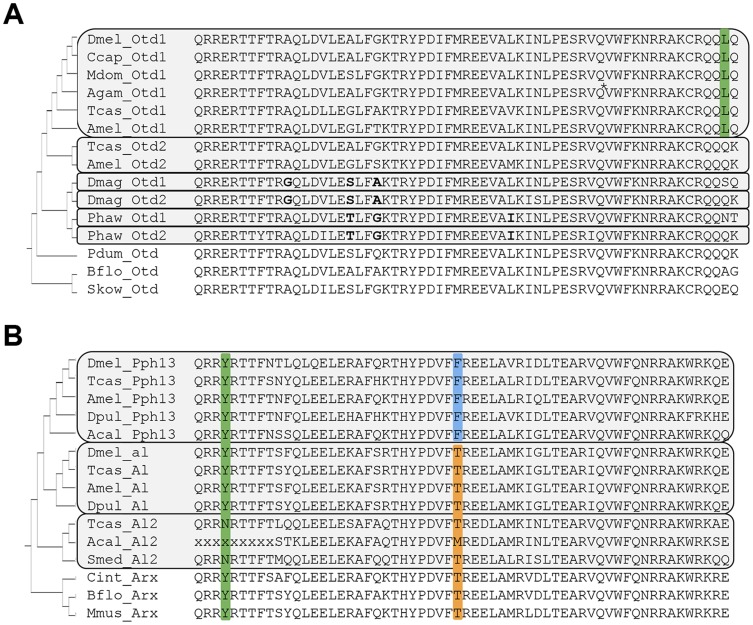
Protein sequence conservation in Otd and Pph13 homologs. A. Otd homologs. Grey boxes outline paralog Otd subfamilies generated by independent gene duplications. The annotation of the *Anopheles gambiae* (Agam) Otd1 contains a putative insertion (VSVGE), which has been removed at the position indicated by asterisk. Green background: Leucine residue diagnostic for insect Otd1. Bold font highlights shared residues in each the *Daphnia* and *Parhyale* Otd duplicates, which support their independent origins in the two lineages. B. Pph13 homologs. Grey boxes outline invertebrate Arx paralog subfamilies generated by independent gene duplications. Green background: Tyrosine (Y) residue diagnostic for Arx gene family members. Blue background: Phenylalanine (F) residue diagnostic for Pph13 subfamily members. Orange background: Threonine (T) residue diagnostic for the Al subfamily and singleton Arx homologs. Species abbreviations: Acal = *Aplysia californica*, Agam = *Anopheles gambiae*, Amel = *Apis mellifera*, Bflo = *Branchiostoma floridae*, Ccap = *Ceratitis capitata*, Cint = *Ciona intestinalis*, Dmag = *Daphnia magna*, Dmel = *Drosophila melanogaster*, Dpul = *Daphnia pulex*, Mdom = *Musca domestica*, Mmus = *Mus musculus*, Pdum = *Platynereis dumerilii*, Phaw = *Parhyale hawaiensis*, Skow = *Saccoglossus kowalevskii*, Smed = *Schmidtea mediterranea*, Tcas = *Tribolium castaneum*.

As a first step towards examining whether the role of Pph13 and Otd in *Drosophila* r- visual photoreceptor differentiation was conserved, we investigated the conservation of orthologs in the genome sequences of the distantly related arthropod species, *Tribolium castaneum* and *Daphnia pulex* [30], [31]. *Tribolium* had been previously shown to possess two paralogs of Otd: Otd1 and Otd2 [32]. The same state was described in the Crustacean *Parhyale hawaiensis* [33]. However, the relationships of the crustacean and coleopteran Otd homologs to the singleton homolog of *Drosophila* were previously considered unresolved due to the low level of sequence conservation outside the homeodomain; within Diptera there has been a reduction to only one *otd* paralog (Figure 1A, S1 and [33]). As in *Parhyale*, our search in *Daphnia pulex* as well as *Daphnia magna* identified two Otd homologs. Protein sequence alignment of an expanded set of Otd homologs (Figure 1A) revealed a highly conserved leucine (L) residue at the C-terminal end of the Otd1 homeodomain, which was unique for Paired-class homeodomain proteins in general [34], distinguished the insect representatives of the Otd1 subfamily, including all dipteran homologs. This finding established *Drosophila* Otd as a member of the insect Otd1 subfamily, implying the loss of insect Otd2 in the evolutionary lineage to Diptera. This conclusion was tentatively supported in a molecular phylogenetic analysis of the relationships between Otd homologs (Figure S1). The latter approach and amino acid residues in the homeodomain that were unique to each of the *Parhyale* and *Daphnia* Otd sequences further suggested that the latter duplicates represented the results of independent gene duplications in crustacean and insect lineages. Thus, the use of the previously introduced acronyms Otd1 and Otd2 for *Parhyale* and *Daphnia* paralogs do not imply specific orthology to insect Otd1 and Otd2.

In contrast to the well-characterized deep conservation of the Otd/Otx gene family, homologs of Pph13 have thus far been identified only in a limited number of insects. Previous efforts identified Pph13 homologs in the *Tribolium* [17] and honey bee genomes [35] but not in the *Daphnia pulex* genome [36]. Closer inspection of candidate *Daphnia pulex* orthologs revealed one annotated locus encoding a 5′ truncated Pph13-related homeodomain, suggesting that the annotation for this locus (JGI_V11_8835 –wfleabase.org) was incorrect. Further examination of upstream genomic regions revealed that the DNA encoding the 5′ portion of the homeodomain was present. This conclusion was confirmed by RT-PCR (data not shown) and in the genome draft of a second related species: *Daphnia magna*. The complete *Daphnia* pulex *Pph13* cDNA sequence thus included sequence from previously annotated loci JGI_V11_8835 and JGI_V11_313449. Sequence conservation between *Daphnia*, *Tribolium*, and *Drosophila* homologs was confined to the homeodomain (Figure 1B). However, examination of a larger sample of Pph13 homeodomain sequences in combination with that of members of the Aristaless (Al/Arx) gene family revealed amino acid residues that defined each subfamily and added further support of their hypothesized common descent (Figure 1B and Figure S2) [35]. A tyrosine (Y) residue at the fourth homeodomain position, otherwise not observed in the *Drosophila* Paired-class homeodomain proteins [34], characterized all members of the Arx gene family (Figure 1B). Furthermore, a phenylalanine (F) residue at homeodomain position 30 specifically marked the Pph13 orthologs, in contrast to the threonine (T) residue in the large majority of Al and Arx orthologs. While this variation was in line with the overall variability at homeodomain position 30, we noted the singularity of the Pph13 characteristic phenylalanine (F) among all *Drosophila* homeodomain proteins [34]. Based on these clues, we also identified putative orthologs of Pph13 and Al in the mollusk *Aplysia californica*. Taken together, these alignment data indicated that a gene duplication at least predating the origin of the Pancrustacea gave rise to Pph13. Finally, we noted the presence of a third Al-related Arx gene family member in the *Tribolium* genome (Tcas A12) that may be of similar ancient origin based on conservation in non-arthropod invertebrates (Figure 1B).

### Pph13 and Orthodenticle are expressed in the developing photoreceptors of *Daphnia* and *Tribolium*

**Figure 2:**
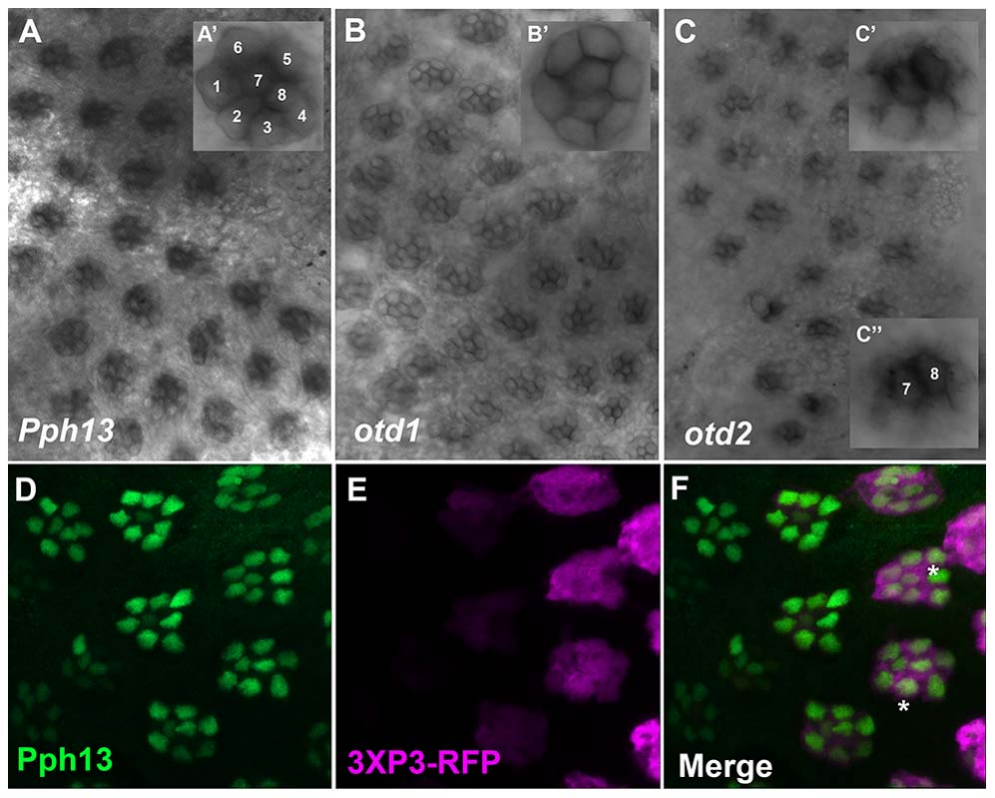
Spatial expression patterns of *Pph13*, *otd1* and *otd2* in the developing adult *Tribolium* visual system. A–C. Dissected retinas 30–40 hrs APF. A. *Pph13* is detected in the developing photoreceptor field and can be observed in all eight photoreceptors of a single ommatidium (A′). B. *otd1* expression is limited to all eight photoreceptors of each developing ommatidium (B′). C. *otd2* expression like *Pph13* and *otd1* is detected in developing photoreceptor field. Expression is greater in the two central photoreceptors R7 and R8 (C″) as compared to the outer six photoreceptors (C′). D–F. Dissected retina *2*4 hrs APF from *Tribolium castaneum* adult visual system stained for Pph13 protein (green) and 3XP3-RFP (magenta). An antibody recognizing Pph13 (green) demonstrates that Pph13 is located in each photoreceptor nucleus. 3XP3-RFP is a cytoplasmic marker for the developing photoreceptors and labels the cell bodies and axon projections of the eight photoreceptors in each ommatidium [25]. Note the expression of Pph13 appears prior to 3XP3-RFP expression (asterisks). In all panels anterior is to the left. Numbers label the eight photoreceptors of a single ommatidium.

The presumed functional conservation of the Pph13 and Otd homologs predicted their expression in photoreceptors of *Tribolium* and *Daphnia*. We therefore assayed the spatial expression patterns of *Pph13*, *otd1* and *otd2* during adult eye development in both *Tribolium* and *Daphnia*. Like *Drosophila*, the *Tribolium* adult compound eye consists of individual ommatidia each of which contains six outer and two inner photoreceptors [37]. Approximately 30–40 hours after pupation we detected a signal from antisense probes to each transcript in all eight photoreceptors of a single ommatidium (Figure 2), whereas the corresponding sense probes did not generate any specific pattern (Figure S3). *Pph13* and *otd1* appeared to have similar expression patterns, with equal levels of transcript in each photoreceptor (Figure 2A and B). An antibody raised against *Tribolium* Pph13 confirmed its expression in the nucleus of each photoreceptor (Figure 2D–F). Together the Pph13 RNA *in situ* pattern and immunofluorescence staining suggested *Pph13* is expressed in every photoreceptor of the eye as observed in *Drosophila*. We also detected *otd2* in all eight photoreceptors but its expression in the two central photoreceptors, R7 and R8, was considerably stronger than in the outer photoreceptors (Figure 2C).

**Figure 3:**
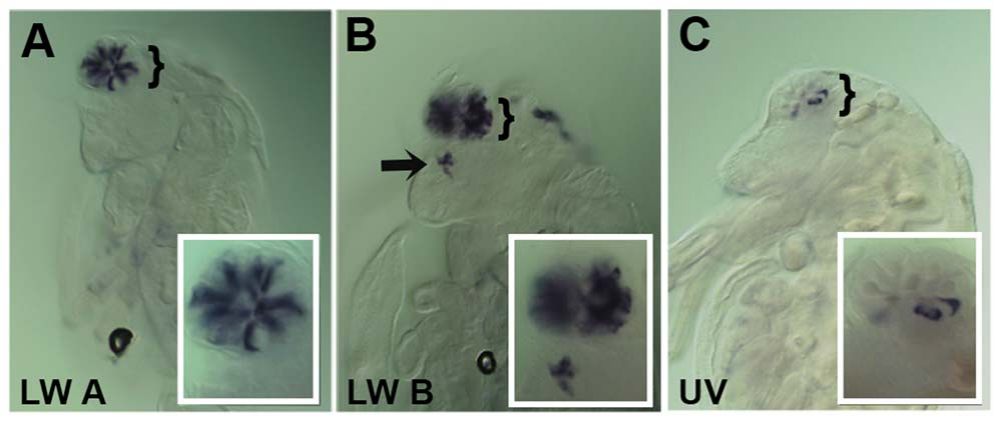
Spatial expression patterns of *Daphnia magna* r- opsins during *Daphnia* eye development. A–C. *Daphnia* embryos 50 hours after egg deposition. A. RNA *in situ* hybridization of a pool containing three antisense probes against potential *Daphnia magna* long wave (LW) clade A r- opsins. B. RNA *in situ* hybridization of a pool containing three antisense probes against potential *Daphnia magna* long wave (LW) clade B r- opsins. C. RNA *in situ* hybridization of an antisense probe against the single potential *Daphnia magna* ultra violet (UV) r- opsin. In all three cases, the probes label photoreceptors in the visual system, and the LW clade B probes also mark and label the photoreceptors of the ocellus (arrow). Insets represent a higher magnified view.

**Figure 4:**
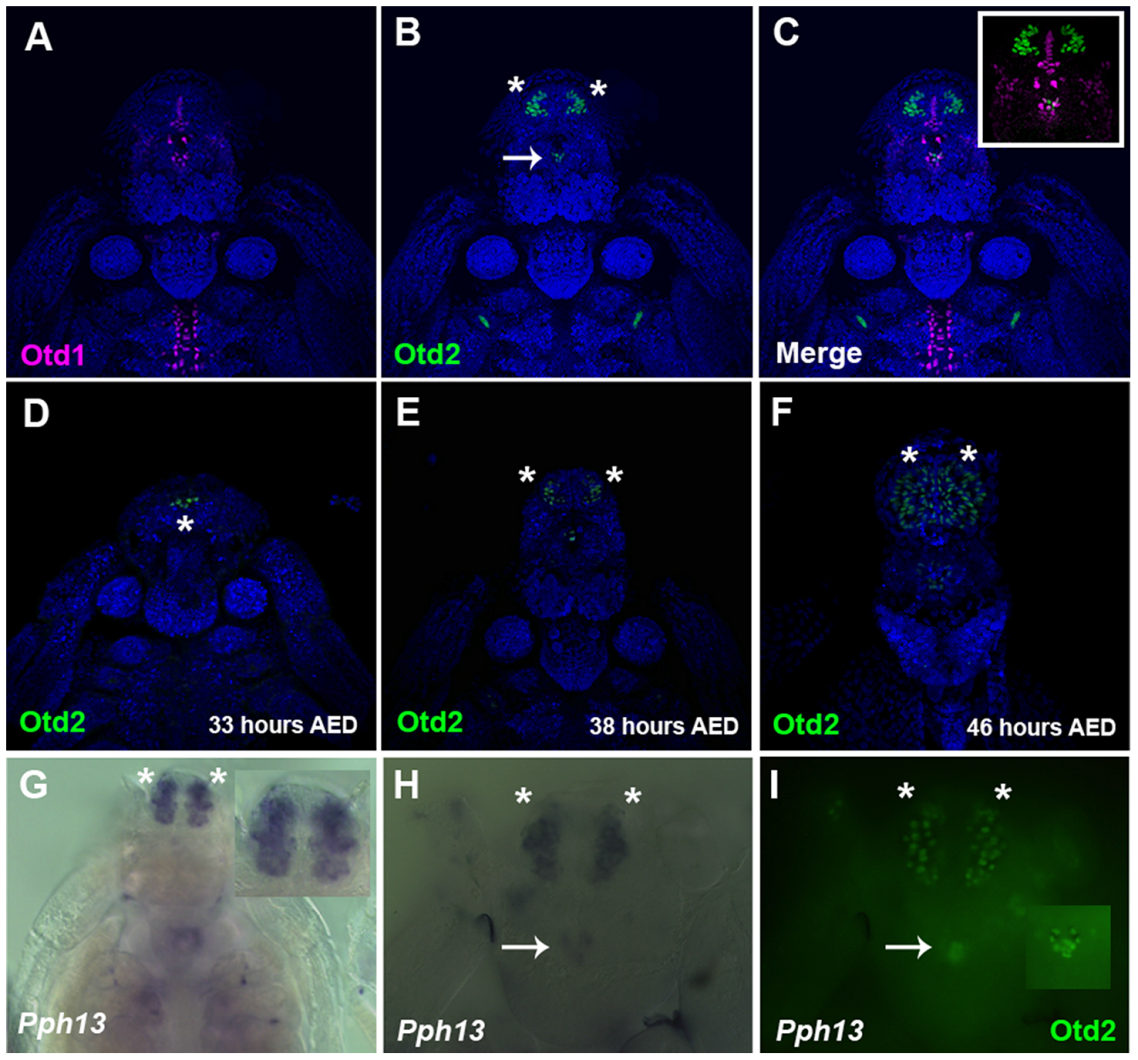
Spatial expression patterns of *Daphnia magna* Otd1, Otd2 and *Pph13* during *Daphnia* eye development. A–C. *Daphnia* embryos 40 hours after egg deposition (AED) stained for Otd1 (magenta) and Otd2 (green) protein. The nuclei were countered stained with DAPI (blue). Otd1 expression is limited to the midline and Otd2 can be detected in the ocellus (arrow) and in the visual system (asterisks). Inset in C is a higher magnification of the head region. D–F. Temporal expression profile of Otd2 expression (green). Otd2 is expressed in an increasing number of positive cells in two lateral symmetrical regions of the head during embryogenesis. G. *Pph13* RNA *in situ* pattern. Inset is a higher magnification of the expression of *Pph13* in the presumptive visual system. H. *Pph13* RNA *in situ* pattern of the same embryo as in I. I. Double label of *Pph13* and Otd2 protein. Note the localization of both Pph13 and Otd2 in the developing eye and ocellus (inset). Asterisks mark the two lateral halves of the developing visual system and arrows mark the ocellus.

In *Daphnia*, the adult eye consists of a single bilaterally symmetrical compound eye, containing a total of twenty-two ommatidia with eight photoreceptors in each ommatidium [38]. Each *Daphnia* eye is generated by the fusion of two lateral groups of ommatidia along the midline late in embryogenesis. Due to the lack of molecular markers, the exact biogenesis of the photoreceptors has not been described. However, previous transmission electron microscopy (TEM) studies of the development of the axonal photoreceptor connections with lamina neurons predict a model in which the photoreceptors begin to differentiate at the midline and move laterally as they mature [39]–[41]. To confirm and differentiate the developing photoreceptors we first examined the expression of a limited set of r-opsins in *Daphnia magna*. In *Daphnia pulex*, there are 27 annotated r-opsin paralogs [30]. Like *Drosophila*, *Daphnia pulex* contain representatives of UV, LW and B- light sensitive r-opsins. This diversity includes 23 LW opsins that split between the LOPA and LOPB clades [30] (Figure S4). The r-opsin family has not been defined for *Daphnia magna* but for our examination we assayed the expression of a pool of three putative representatives from LOPA (Figure 3A) and LOPB (Figure 3B) and the putative UV opsin (Figure 3C). Besides observing a differential display of expression between all three groups, the RNA *in situ* hybridization patterns confirmed and delineated the embryonic tissue that gives rise to the photoreceptors of the eye and ocellus (Figure 3 and Figure S5A–F). Our expression analysis of Otd1, Otd2, and *Pph13* in *Daphnia magna* also yielded results that were consistent with this model of eye formation (Figure 4 and Figure S5G–J). The expression of Otd1 was limited to the midline region of the embryo and not necessarily associated with visual photoreceptors (Figure 4A,C). In contrast to Otd1, Otd2 was expressed in an increasing number of cells in two lateral symmetrical regions of the head during embryogenesis (Figure 4B–F). By 48 hours after egg deposition (AED), each lateral cluster contained approximately 75–78 Otd2 positive cells (Movie S1). Considering that the adult eye consists of two lateral clusters of eleven ommatidia, this suggested that there were seven Otd2 positive photoreceptors per ommatidium. Furthermore, the additional Otd2 staining present in the central portion of the embryo corresponded to the region where the ocellus develops (Figure 4A–F and Figure 3B). Similar and consistent expression patterns were detected for *Daphnia magna* homolog of *Pph13*, which was expressed in two symmetrical lateral clusters of cells, in the cells of the presumptive ocellus (Figure 4G–I) potentially colocalizing with Otd2 protein expression (Figure 4H,I).

### RCSI-like sites are present in the promoters of r-opsin genes of *Tribolium* and *Daphnia*

To further explore the possibility of conserved regulatory roles of Pph13 and Otd in visual photoreceptor differentiation, we probed for the conservation of the RCSI site in candidate target genes of Pph13 in *Tribolium* and *Daphnia*. Previous work has shown that Pph13 binds a subset of RCSI sites and that this binding site is essential for transcriptional activation [19], [20]. The same studies defined a Pph13 RCSI site as a palindromic sequence of TAAT spaced by three nucleotides with one half site matching the consensus sequence of 5′-CTAATTG-3′ [20]. In *Drosophila*, these Pph13-specific RCSI sites are present in the 5′ cis-regulatory DNA of r- opsin genes and in several other key phototransduction proteins, including the heterotrimeric G-protein β subunit (Gβ76C) [19], [20]. *Tribolium* contains two r- opsins, one of which belongs to the LW opsin subfamily and one of which belongs into the UV sensitive subfamily [42]. Scanning their upstream regions for the RCSI motif, we found a potential RCSI site in both of them. Furthermore, in the upstream region of the *Tribolium* homolog of *Drosophila* visual Gβ (Gβ76C), LOC662674 (beetlebase.org), we also detected a RCSI site (Table S1 and Figure S6). Examining the immediate upstream regions of *Daphnia* LW, UV, and B opsins revealed putative RCSI motifs only in the LOPB clade of LW opsins (Figure S4 and Table S1). In addition, the closest homolog to both *Drosophila* and *Tribolium* visual Gβ subunit in *Daphnia* (JGI_V11_210534 -wfleabase.org) contained a potential RCSI site (Figure S6 and Table S1).

### Conserved binding specificity of Pph13 homologs to *Tribolium* and *Daphnia* RCSI sites

**Figure 5:**
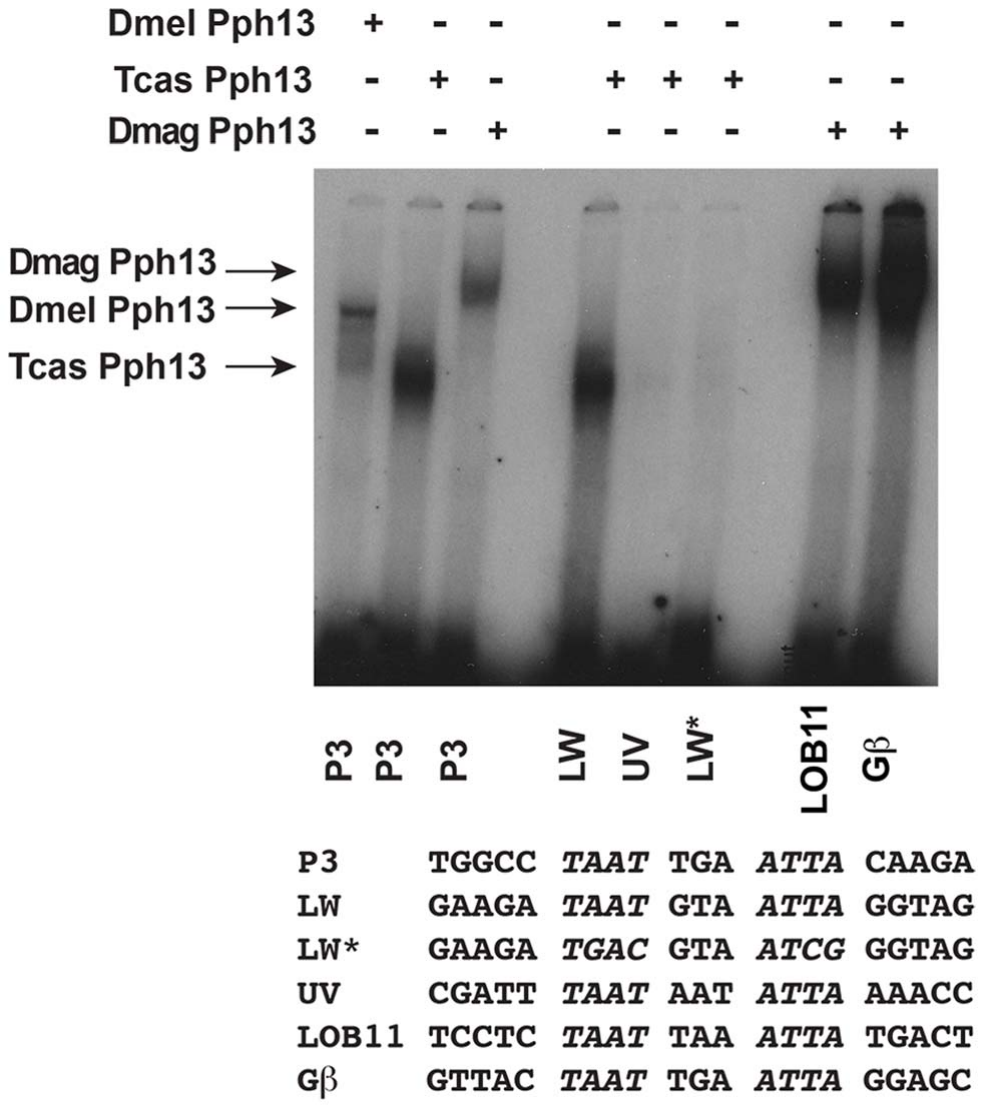
DNA binding properties of Pph13 are conserved among Pancrusteceans. Electrophoretic mobility shift assays of *Drosophila* (Dmel) and *Tribolium* (Tcas) and *Daphnia magna* (Dmag) Pph13 protein on a Pax6 homeodomain binding site (P3) and on endogenous *Tribolium* and *Daphnia* RCSI sites. Each homolog has the ability to bind the P3 consensus binding site as well as endogenous sites within its genome. *Tribolium* Pph13 demonstrates a differential binding to the RCSI sites identified in the cis-regulatory regions of the *LW* and *UV* r- opsins and binding is disrupted upon the mutation of the LW RCSI site (LW*). Arrows indicate the specific mobility shift for each protein examined.

The presence of potential RCSI sites in photoreceptor-expressed genes of all three species suggested that these sites could serve as Pph13 binding sites. To test this possibility, we investigated whether the Pph13 homologs could bind the putative endogenous RCSI sequences with electrophoretic mobility shift assays (EMSAs). These experiments revealed that *Tribolium* and *Daphnia* Pph13 have similar binding abilities to a consensus RCSI site (P3) [24], [43] as well as specific *Drosophila* RCSI sites (Figure 5 and data not shown). Furthermore, each has the capability to bind to their endogenous RCSI sites (Figure 5 and Figure S7). Interestingly, in *Tribolium* like *Drosophila* [20], we observed a differential affinity of Pph13 to the identified RCSI sites of UV and LW r-opsins. *Tribolium* Pph13 bound efficiently to the LW opsin RCSI site but binding was barely detectable on the UV opsin RCSI element, suggesting that the simple presence of a correctly spaced palindromic sequence of TAAT was not sufficient to bind Pph13.

### Equivalent *in vivo* function of Pph13 homologs

The *in vivo* expression patterns and *in vitro* binding assays provided strong evidence that Pph13 and Otd regulate r- visual photoreceptor cell differentiation in *Drosophila*, *Tribolium* and *Daphnia*. To test for functional equivalency among the orthologs and, more importantly, the ability to direct photoreceptor differentiation, we examined whether *Daphnia* and *Tribolium* orthologs were capable of rescuing the photoreceptor defects observed in *Drosophila Pph13* and *otd* mutants.

**Figure 6:**
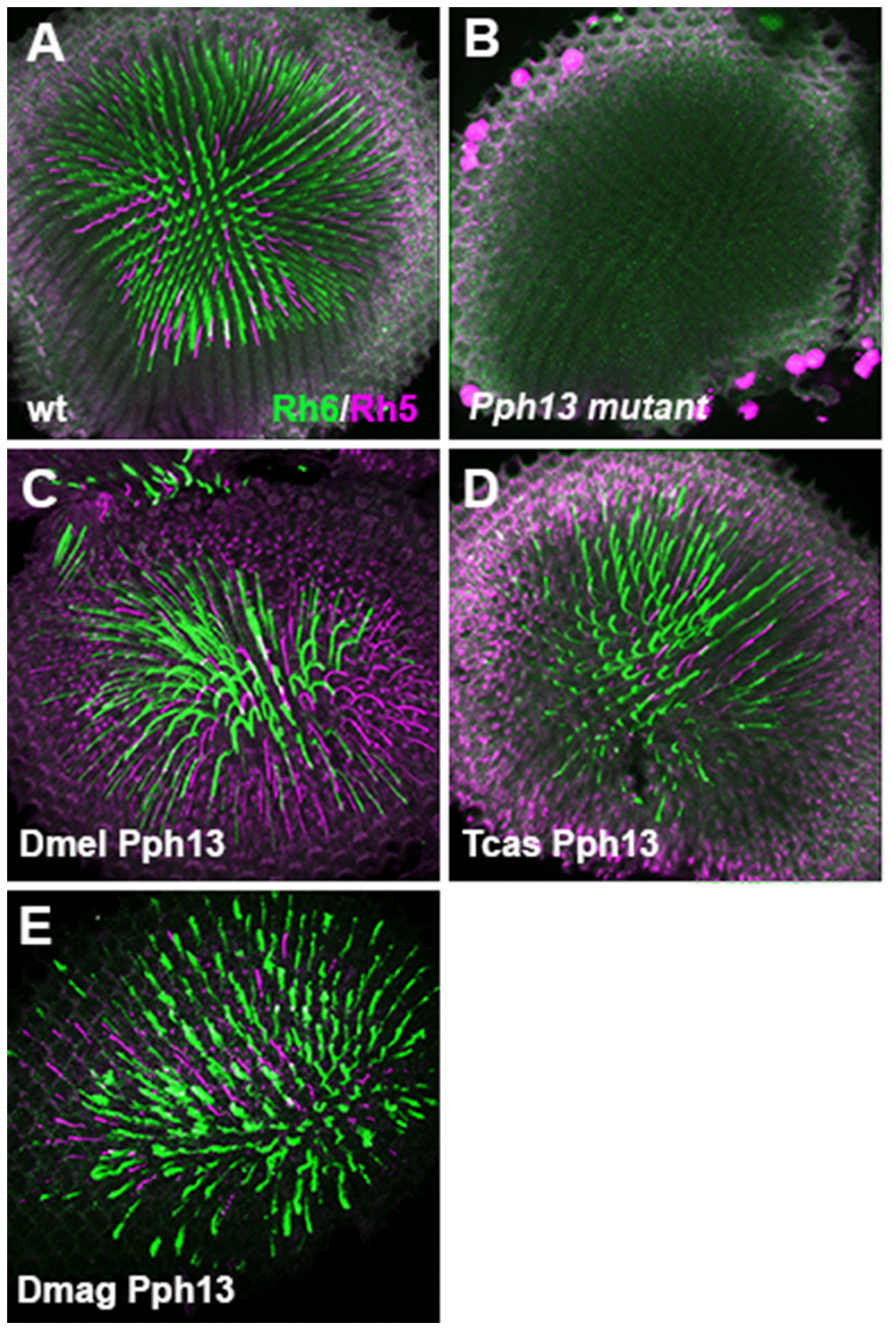
*In vivo* rescue of opsin expression in *Pph13* null mutant *Drosophila*. A–E. Rh6 (green) and Rh5 (magenta) opsin protein expression in adult *Drosophila* retinas. A. Wild-type retina. Opsin protein accumulates in the rhabdomeres and thus appears as a tube like structure. Rh6 and Rh5 are expressed a non-overlapping subsets of central R8 photoreceptors. B. *Pph13* mutant. Neither Rh6 nor Rh5 opsin can be detected, reflecting the facts that *Rh6* is a direct transcriptional target of Pph13 and that the deformed development of rhabdomeres in *Pph13* mutants prevents the accumulation of detectable levels of Rh5 opsin. C–E. Rescue of the *Drosophila Pph13* mutant with: C. *Drosophila* (Dmel) *Pph13*. D. *Tribolium* (Tcas) *Pph13*. E. *Daphnia magna* (Dmag) *Pph13*. In all rescue experiments we observe the restoration of Rh6 and Rh5 protein expression in the rhabdomeres. Furthermore, the non-overlapping expression of Rh6 and Rh5 in R8 photoreceptors is likewise rescued. Each panel represents a projection of a confocal stack of laser scanning confocal microscope images.

**Figure 7:**
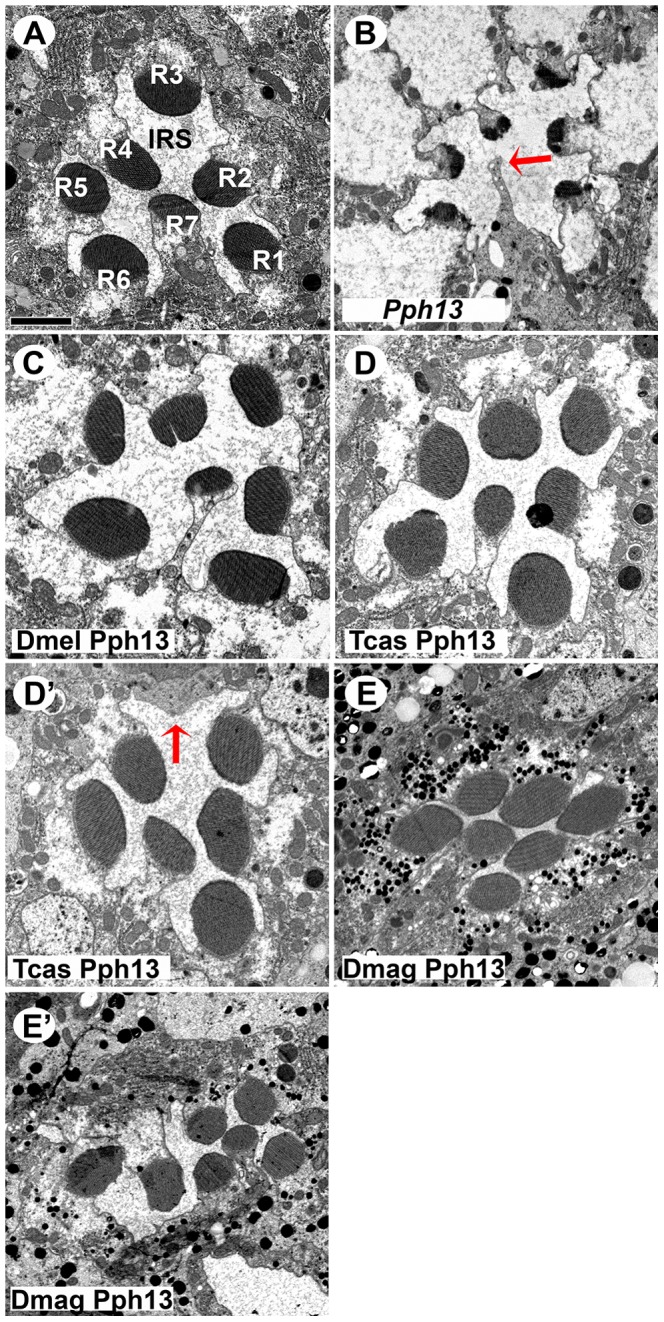
*In vivo* rescue of rhabdomere morphology in *Pph13* null mutant *Drosophila*. A–E. Transmission electron microscopy analysis of adult *Drosophila* eyes. A. Wild-type ommatidium. Seven of the eight photoreceptors and associated rhabdomeres (R1–R7) can be observed per section and each rhabdomere is separated by an inter-rhabdomeral space (IRS). B. *Pph13* mutant. The absence of Pph13 activity results in the reduction, malformation or loss of rhabdomeres (arrow). C–E. Rescue of the *Drosophila Pph13* mutant with: C. *Drosophila* (Dmel) *Pph13*. Rhabdomere morphology is restored. D. *Tribolium* (Tcas) *Pph13*. Rhabdomere morphology is restored but occasionally a missing rhabdomere is observed (D′). The arrow denotes a missing rhabdomere of a photoreceptor but the photoreceptor is present. E. *Daphnia magna* (Dmag) *Pph13*. Whereas apical sections (E) demonstrate complete restoration of rhabdomere morphology, basal sections (E′) demonstrate that many rhabdomeres do not extend completely or maintain their shape throughout the length of the photoreceptor. Scale bar 2 um.

*Drosophila Pph13* mutants have two distinct characteristics. First, Pph13 is necessary for expression of opsin *rh6* (Figure 6A,B) in 70% of the R8 photoreceptor cells [44]. Second, *Pph13* mutants have severe defects in rhabdomere morphology (Figure 7A,B). The morphological defects are acute enough to hamper the detection and accumulation of other r-opsins [20], [21] (Figure 6B). For rescue experiments, each homolog was placed under the control of GAL4 transcription [45] and inserted into the identical locus in the *Drosophila* genome. To drive expression, we generated a GAL4 driver under the control of the endogenous *Drosophila Pph13* cis-regulatory region – *Pph13*-Gal4. In testing the Pph13 homologs of *Daphnia* and *Tribolium*, we evaluated both the restoration of Rh6 opsin expression and rhabdomere morphogenesis as compared to rescue with *Drosophila* Pph13. We found that all Pph13 homologs were capable of restoring Rh6 expression. In addition, we also detected a mosaic expression pattern of Rh6 in the R8 photoreceptors (Figure 6C–E). Of note, this result also demonstrated the specific rescue of rhabdomere morphology in the R8 photoreceptors that express Rh5 opsin in a Pph13 independent manner. To assay rhabdomere morphology directly, TEM analysis of each rescue condition was performed. We observed wild-type rescue of rhabdomere morphology with all three Pph13 homologs (Figure 7). However, the rescue was not fully penetrant with *Tribolium* and *Daphnia* Pph13. In particular, we observed photoreceptors missing rhabdomeres with *Tribolium* Pph13 (Figure 7D′) and with *Daphnia* Pph13, the rhabdomeres did not maintain their position and morphology along the proximodistal axis of the photoreceptor (compare Figure 7E and E′).

### Orthodenticle orthologs demonstrate differential abilities with respect to photoreceptor differentiation

For examination of functional equivalency among Otd orthologs, we used a previously established rescue paradigm [46], [47]. Otd is required for *rh3* opsin expression in the distally located inner photoreceptor, the activation of *rh5* in the proximal inner photoreceptor, repression of *rh6* opsin in the outer photoreceptors, and correct rhabdomere morphology in every photoreceptor [13], [15], [16]. In our rescue experiments, we assayed for all these functions.

**Figure 8:**
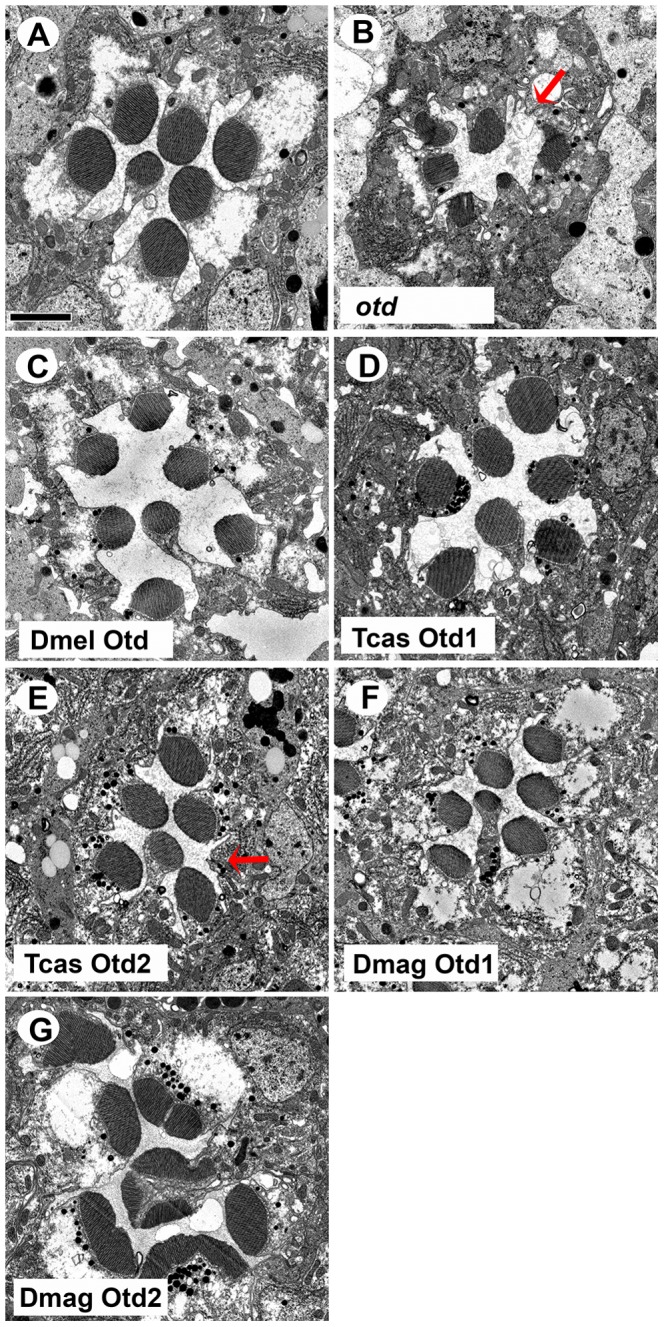
*In vivo* rescue of rhabdomere phenotype of *Drosophila orthodenticle* mutant background. A–G. Transmission electron microscopy analysis of adult *Drosophila* eyes. A. Wild-type ommatidium. B. *otd* mutant. The absence of Otd activity results in smaller malformed or even absent rhabdomeres (arrow). C–G. Rescue of *otd* mutant with: C. *Drosophila* (Dmel) *otd*. D. *Tribolium* (Tcas) *otd1*. E. *Tribolium* (Tcas) *otd2*. The arrow denotes a missing rhabdomere of a photoreceptor. F. *Daphnia magna* (Dmag) *otd1*. G. *Daphnia magna otd2*. In all cases, except Dmag *otd2*, rhabdomere morphology is restored. The rhabdomere phenotype observed with Dmag *otd2* differs from both wild type and the *otd* mutant phenotype. Scale bar 2 um.

With respect to rhabdomere morphology (Figure 8), TEM analysis demonstrated that both *Tribolium* Otd paralogs (Figure 8D,E) and *Daphnia* Otd1 (Figure 8F) could direct rhabdomere morphogenesis in *Drosophila*. In these three rescue experiments, we observed the return of symmetrical wild-type pattern of rhabdomere shape and size. However, the rescue was not fully penetrant with *Tribolium* Otd2 and *Daphnia* Otd1. For example, some ommatidia have photoreceptors that lack a detectable rhabdomere. The expression of *Daphnia* Otd2 resulted in an adult eye that contained a mosaic of intact and dead tissue, regardless of the presence of endogenous Otd (Figure S8); this phenotype was not observed with any of the other Otd orthologs. TEM analysis confirmed the lack of photoreceptors in the discolored regions (data not shown). In the normal pigmented regions we observed the presence of rhabdomeres that were not characteristic of the *otd* mutant or normal rhabdomeres in *Drosophila* suggesting these defects resulted from the misexpression of the non-endogenous Otd (Figure 8G), which prevented further analysis.

**Figure 9:**
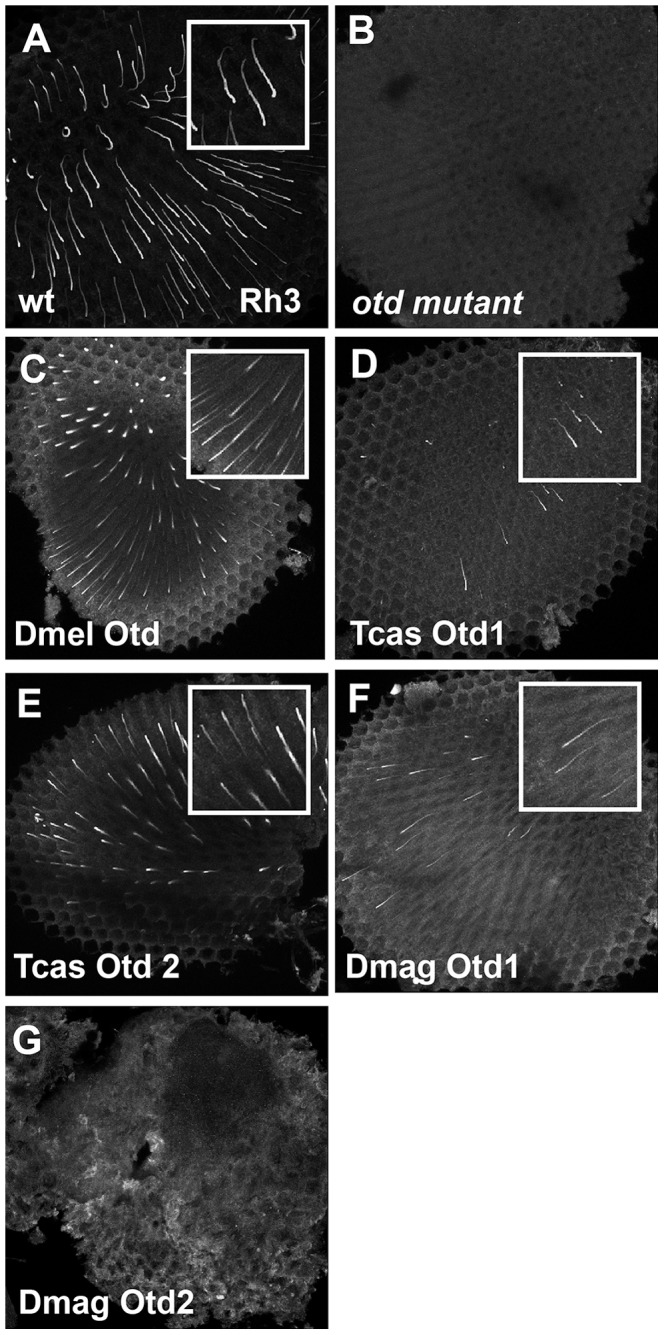
*In vivo* rescue of Rh3 opsin expression in *Drosophila orthodenticle* mutant background. A–E. Rh3 opsin protein expression in adult *Drosophila* retinas. A. Wild-type retina. Rh3 opsin protein accumulates in the rhabdomeres of approximately 30% of the R7 photoreceptor cells. B. *otd* mutant. Consistent with the requirement of Otd for the transcriptional activation of *rh3*, no protein is detected. C–G. Rescue of *otd* mutant with: C. *Drosophila* (Dmel) *otd*. D. *Tribolium* (Tcas) *otd1*. E. *Tribolium* (Tcas) *otd2*. F. *Daphnia magna* (Dmag) *otd1*. G. *Daphnia magna otd2*. All Otd orthologs are capable of restoring Rh3 expression in an *otd* mutant except Dmag *otd2*. Insets represent a magnified view of a region from each panel. Each panel represents a projection of a confocal stack.

As for rescue of r- opsin regulation (Figure 9 and Figures S9 and S10), our results exposed differential effects among the Otd orthologs. For example, *Tribolium* Otd1 could activate *rh3* expression (Figure 9D) but failed to repress *rh6* expression (Figure S9D). *Tribolium* Otd2 in contrast was capable of both (Figure 9E and S9E). On the other hand, *Daphnia* Otd1 could activate *rh3* expression and repress *rh6* expression (Figure 9F and S9F), even though *Daphnia* Otd1 does not appear to be expressed in photoreceptors. Despite the associated cell death and irregular rhabdomere morphology with expression of *Daphnia magna* Otd2, we observed that the expression of Rh6 appeared to be limited to a single photoreceptor of each ommatidium suggesting *Daphnia magna* Otd2 can execute the *rh6* repression function (Figure S9G). However, we did not detect any expression of Rh3, suggesting that *Daphnia magna* Otd2 failed to execute the *rh3* activation function (Figure 9G). In *Drosophila*, the expression of opsin *rh5* in a subset of R8 photoreceptors is dependent on direct activation by Otd. Moreover, Otd also regulates the feedback loop responsible for generating the correct ratio of Rh5 and Rh6 expressing R8 photoreceptors [16], [48]. In agreement with previous results utilizing this rescue paradigm *Drosophila* Otd is relatively insufficient in activating *rh5* expression [47] and only detected Rh5 expression upon the rescue with *Tribolium* Otd2 (Figure S10).

### *In vivo* requirements of Pph13 and Orthodenticle in *Tribolium* rhabdomere formation

**Figure 10:**
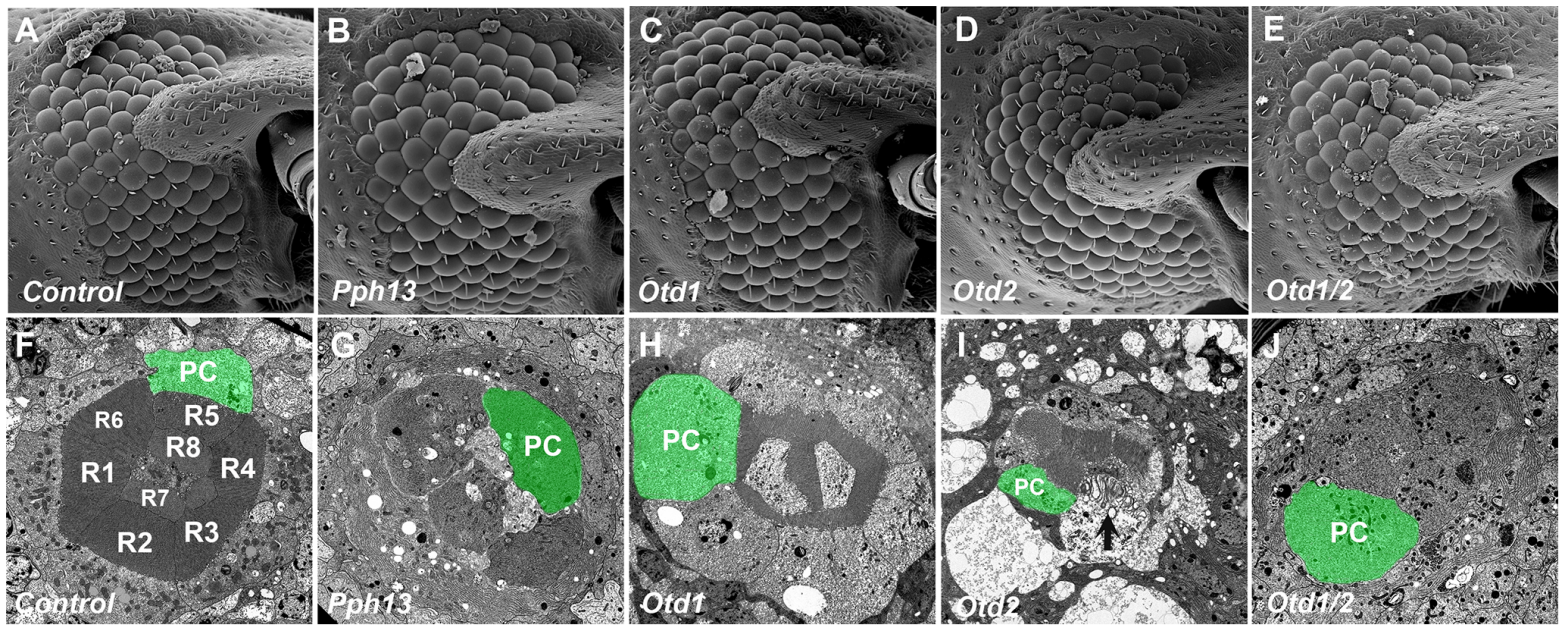
Knockdown of *Tribolium Pph13*, *otd1* and *otd2* affects rhabdomere biogenesis. Scanning and transmission electron microscopy analyses of *Tribolium* adult eyes. A,F. Mock injection. B,G. *Pph13* RNAi. C,H. *otd1* RNAi. D,I. *otd2* RNAi. E,J. *otd1* and *otd2* RNAi. The green shading marks the cell body of one of the eight photoreceptors (PC) found in each ommatidium. Each photoreceptor produces a rhabdomere (R1–R8) that remains juxtaposed to the others. The knockdown of *Pph13* and *orthodenticle* paralogs did not affect the overall structure of the adult eye (A–E). However, the knockdown of *Pph13* (G) and the combinatorial removal of both *otd* paralogs result in a complete absence of rhabdomeres (J). Photoreceptors are detected but the microvillar rhabdomere structures are absent. The knockdown of *otd1* generates smaller rhabdomeres (H) and the removal of *otd2* (I) alone results in rhabdomeres in an active state of degeneration (arrow).

Taken together, our expression and rescue assays supported a common role of Pph13 and Otd in r- visual photoreceptor differentiation among Pancrustaceans. For further functional verification, we took advantage of the effective RNAi protocol in *Tribolium* [49]. Thus to examine the *in vivo* role of the *Tribolium Pph13*, *otd1*, and *otd2* genes, we generated double stranded RNA (DsRNA) against each corresponding mRNA for injection into *Tribolium* larvae. None of the DsRNAs affected developmental timing, viability, or external morphological structures as compared to mock injections; scanning electron microscopy of the adult eye did not reveal any major effects on the external organization of the compound eyes (Figure 10A–E). To investigate the possible role of Pph13 and Otd in rhabdomere morphogenesis, RNAi knockdown adults were prepared for TEM analysis within twelve hours after eclosion to minimize any potential later disruptions of photoreceptor morphology as a result of long-term degeneration. In these specimens, we found that the knockdown of *Tribolium Pph13* resulted in the complete absence of rhabdomeres (Figure 10G). The knockdown of either *Tribolium otd1* or *otd2* caused a completely distinct set of defects. In *otd1* knockdowns, the rhabdomeres were present but reduced compared to wild-type controls (Figure 10H). The ordered array of microvillar projections was normal in each of the rhabdomeres but the microvilli were smaller but no evidence of photoreceptor degeneration could be detected. In *otd2* knockdown animals, the rhabdomeres were in a state of disarray suggesting degeneration (Figure 10I and Figure S11). We detected whole rhabdomeres that however appeared to be unraveling; as suggested by the presence of large membrane protrusions into the photoreceptor cell body and enlarged distances between microvillar projections. In addition some photoreceptors completely lacked rhabdomeres. Lastly, the combinatorial removal of both *otd* paralogs resulted in the absence of all rhabdomere structures (Figure 10J).

### Differential requirements of Pph13 and Orthodenticle for r- opsin expression in *Tribolium*

**Figure 11:**
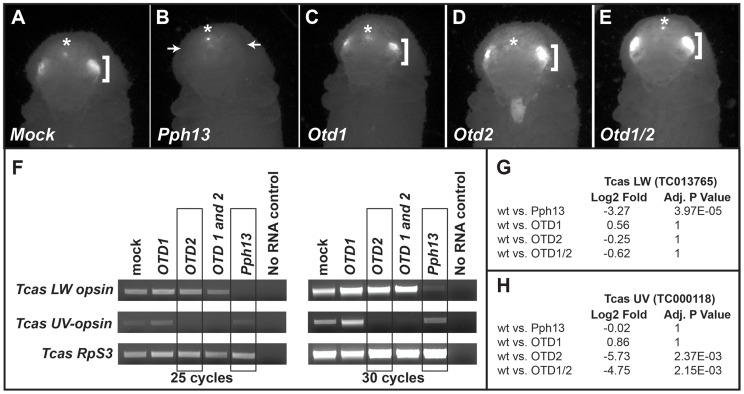
*Tribolium* Pph13 and Otd2 are required for r-opsin expression. A–E. 3XP3-RFP expression in RNAi knockdown animals. Photoreceptor specific RFP fluorescence can be detected through the pupal case in the developing adult eyes (brackets). Only the knockdown of *Pph13* results in the loss of the transcriptional reporter 3XP3-RFP fluorescence (arrows). The asterisks mark the non-visual enhancer trap expression of RFP that is present in each RNAi knockdown animal. F. RT-PCR analysis of r-opsin transcription in *Tribolium* (Tcas) *Pph13*, *otd1*, *otd2*, and *otd1/2* RNAi knockdown animals. The transcriptional levels of *LW* r-opsin are only reduced/absent with the reduction of *Pph13* and transcription levels of *UV* r- opsin are only affected by the reduction of *otd2*. The transcription of *Tribolium* ribosomal protein subunit three (*RpS3*) was used as a control for comparisons. G–H. RNA-seq quantification of r- opsin expression levels, *LW* (G) and *UV* (H) in the knockdown animals. The RNA-seq data confirms the requirement of Pph13 and Otd2 for transcription of r- opsin *LW* (TC013765) and *UV* (TC000118), respectively. Wild type (wt) are represented by the control mock injections. P-Values have been adjusted for multiple testing.

As a first approximation of whether Pph13 and Otd homologs may be required for the second step defining r- visual photoreceptor differentiation, i.e. the transcription of the phototransduction machinery, we asked if 3XP3-RFP, an *in vivo* transcriptional reporter for Pph13 activity in *Drosophila* [20], was disturbed upon reduction of *Pph13*, *otd1* or *otd2* (Figure 11). DsRNAs against each transcript were injected into *Tribolium m26* larvae, which express RFP from a 3XP3-RFP reporter transgene. In these experiments, only the *Pph13* knockdown resulted in the elimination of the photoreceptor specific expression of RFP (Figure 11B and Table S2). Single as well as combinatorial injection of the *Tribolium otd1* and *otd2* dsRNAs did not affect the expression of 3XP3 reporter (Figure 11C–E).

To further explore whether *Tribolium* Pph13 and Otd were required for r- opsin expression in *Tribolium*, we assayed the transcription of the *Tribolium LW* and *UV* opsins by RT-PCR (Figure 11F) and by RNA-seq (Figure 11G,H and Table S5 and S6) in the RNAi knockdown conditions. Based on our DNA binding assays and the requirement of Pph13 for 3XP3 expression, we predicted that the knockdown of *Pph13* should affect only *LW* r- opsin transcription. Indeed, the knockdown of *Tribolium Pph13* was associated with the reduction of *LW* transcription but not *UV* opsin transcription (Figure 11F,G). There was a 3.27 Log2 fold decrease in *LW* opsin expression in *Pph13* knockdown animals as compared to mock injections. The knockdown of *Otd2*, however, resulted in the virtual absence of *UV* opsin transcription (Figure 11F,H), as indicated by 5.73 Log2 fold decrease compared to control animals. Finally, DsRNA directed against both *Tribolium otd* paralogs had no discernible effect on *LW* opsin transcription (Figure 11F,G). Although *Tribolium otd1* is expressed in all photoreceptors, we did not detect any significant effect on *UV* expression alone or enhancement in combination with knockdown of *otd2* (Figure 11F,H).

## Discussion

### The photoreceptor-specific function of Pph13 extends to the ancestor of Pancrustaceans

Our results demonstrate a key role of both Pph13 and Otd for visual r- photoreceptor differentiation among Pancrustaceans. While the conservation of Otx transcription factors in metazoan eye development has been documented by numerous studies [47], [50]–[53], our data provide the first evidence of a deeper evolutionary conservation of Pph13. In combination with the initial failure to detect orthologs in other species beyond *Drosophila*, the specificity of Pph13 expression and function to a single developmental context, terminal photoreceptor differentiation and maintenance [19], [20], raised the possibility that Pph13 represented a more recently evolved regulator in insect retinal development; our findings here refute this scenario. Moreover, our comparative sequence analyses consolidate that Pph13 arose by duplication of an ancestral singleton member of the Arx gene family of homeodomain transcription factors, as previously hypothesized [18], [19]. Further identification of orthologs will be required to date the exact time point of this gene duplication. In our preliminary analyses we have also identified putative Pph13 and Al orthologs in the mollusk *Aplysia califonica*. However, searches in genome and transcriptome data from other invertebrates as well as non-Pancrustacean arthropods recovered only orthologs of Al/Arx at this point.

Here, in this study, we have deployed a combination of assays to assess the functional conservation of Pph13 over hundreds of millions of years of Pancrustacean evolution. Our expression analyses revealed that Pph13 is specifically expressed in the visual systems of *Daphnia* and *Tribolium*. Furthermore, this study and previous work now identifies the binding of Pph13 to the RCSI site as the driver of default activation of LW-opsins in both *Drosophila* and *Tribolium*. Our findings demonstrate the conserved ability of Pph13 homologs to discriminate between RCSI sites, preferably binding the RCSI sites in LW r-opsins [20]. Moreover, the functional analysis in both *Drosophila* as well as *Tribolium* reveals that Pph13 is required for transcription of only the LW r- opsins. We therefore believe that activation through Pph13 at the RCSI site was a module in the cis-regulatory control of the ancestral singleton LW-opsin. Consistent with the conservation of this evolutionarily conserved target sequence, all three homologs are capable of rescuing both aspects of r- photoreceptor differentiation in *Drosophila Pph13* mutants: rhabdomere biogenesis, and opsin regulation. The observed decrease in Pph13 rescue efficiency with evolutionary distance may be the result of a failure to interact with the required cofactors in *Drosophila* or to activate transcription through the *Drosophila* RCSI sites. Given the conserved binding activity of all Pph13 homologs, it is most likely that the lack of sequence conservation outside the homeodomain compromises interactions with cofactors in the across-species rescue experiments.

### Diversified subfunctionalization of Otd paralogs in rhabdomeric photoreceptor differentiation

Our data also demonstrate conserved critical roles of Otd in both aspects of rhabdomeric photoreceptor differentiation. However, the presence of two paralogs in *Tribolium* and *Daphnia*, and their differential expression patterns and functional abilities complicate defining the ancestral role of Otd in visual r- photoreceptor differentiation. In all three species at least one Otd ortholog is expressed in developing photoreceptors. Further, the downregulation of Otd orthologs leads to a disruption of rhabdomere formation in both *Drosophila* and *Tribolium*. Moreover, with the exception of *Daphnia magna* Otd2, each Otd homolog that we tested has maintained the ability to direct rhabdomere morphogenesis in *Drosophila*. The expression of *Daphnia magna* Otd2 in *Drosophila* resulted in cell death and as such precluded assessment of its ability to rescue the *Drosophila otd* mutant. Taken together, these data suggest that the requirement of Otd in rhabdomere morphogenesis is ancestral. In agreement with this, previous studies demonstrated that all three vertebrate OTX paralogs were capable of rescuing rhabdomere morphogenesis when expressed in *Drosophila otd* mutant photoreceptors [47].

Consistent with the evidence of independent duplication events in the insect and crustacean lineages, we find that there is no simple correlation between the expression profiles of *otd* paralogs and their ability to direct r- opsin expression in our data set. Most conspicuously, *Daphnia magna* Otd1, which is not detected in photoreceptor cells within *Daphnia magna*, has the ability to execute both the repression of *rh6* and activation of *rh3* r- opsins in the *Drosophila* Otd rescue paradigm. *Daphnia magna* Otd2, on the other hand, which is endogenously expressed in photoreceptor cells, was only able to rescue the appropriate repression of *Drosophila rh6*, which however works in conjunction with defective proventriculus (Dve) [48]. It is also noteworthy that while we could not establish that *Daphnia* Otd1 is orthologous to *Drosophila* Otd or *Tribolium* Otd1, it has the ability to rescue both *rh6* repression and *rh3* activation whereas *Tribolium* Otd1 the ortholog of *Drosophila* Otd rescued only the activation of *rh3*. Moreover, even though *Tribolium* Otd1 is expressed in all photoreceptors and has the ability to activate r- opsin transcription in *Drosophila*, this ortholog is apparently dispensable for r- opsin expression within *Tribolium*. Interestingly, the three vertebrate Otx homologs also exhibited differential rescuing activities of *rh3*, *rh5* and *rh6* regulation [47]. The sum of these data indicates that the paralogs, which originated through independent duplications of the *otd* locus in insect and crustacean lineages contributed to different subfunctionalization trajectories. While the functional diversification that resulted from this appears bewildering, it implies continued evolutionary interchangeability, which may have been key to consolidating all Otd-related functions onto a single homolog during the loss of *otd2* in the lineage to dipteran species.

### Evidence of evolutionarily diversified target gene distribution between Pph13 and Otd with respect to rhabdomere biogenesis

The activation of structural gene batteries forms the endpoint in the gene regulatory network control of cell differentiation [54]. The synergistic activation of the structural genes by both Otd and Pph13 in the *Drosophila* eye is a good example of this paradigm. Interestingly, our functional analysis in *Tribolium* reveals differences in how Pph13 and Otd are employed in directing rhabdomere morphogenesis compared to *Drosophila*. First, within *Tribolium*, the reduction of either Otd1 or Otd2 generates non-overlapping defects in rhabdomere morphogenesis while the simultaneous knockdown of both genes leads to complete failure of rhabdomere formation. This outcome could result from incomplete subfunctionalization, leaving a limited degree of genetic redundancy in place. Alternatively, the two paralogs may have limited capacities to compensate for the downregulation of the sister paralog via expression level increase. Second, in *Drosophila*, both Pph13 and Otd are providing independent and overlapping functions to generate the rhabdomere [20]. As a result, the removal of both Otd and Pph13 is required to generate photoreceptors that lack rhabdomeres. In *Tribolium*, the knockdown of either *Pph13* alone or the knockdown of both *otd* paralogs is sufficient to eliminate rhabdomeres. Thus, there appears to be significantly less redundant control of rhabdomere formation in *Tribolium* in contrast to *Drosophila*. Assuming the general conservation of rhabdomere structure target genes, this finding implies evolutionary turnover of Otd and Pph13 dedicated target genes in the context of rhabdomere formation. Given the well defined binding properties of Pph13 and Otd and the conservation of the Pph13 binding sites between *Drosophila* and *Tribolium*, it should be feasible to elucidate the diverged distribution of Otd versus Pph13 targets in *Tribolium* versus *Drosophila*. Such studies will expand our understanding of the evolution of gene regulatory networks by specifically testing the proposal that downstream network components enjoy greater degrees of evolutionary freedom than intermediate upstream components [55].

### Ancestral and regulatory switches driving opsin expression and morphogenesis in both rhabdomeric and ciliary photoreceptors

Ever since Darwin pondered about the evolution of the eye [56], the process has and continues to be a challenge to investigators [57]. To date, with respect to photoreceptors, it is now accepted that the two fundamental types, ciliary and rhabdomeric, were present before the split of bilaterian animals and share a common ancestor [3]–[6]. However, compounding the study of the evolution of photoreceptors is the fact that the photoreceptors are utilized in various visual and non-visual light detection systems and obtain diverged morphological states in both protostomes and deuterostomes. Therefore, a critical component to provide clarity for defining homologous photoreceptors [3] is to identify the conserved regulatory proteins and switches required for the differentiation of the various classes of animal photoreceptors. Our studies have now identified two common regulators of one type of photoreceptor: rhabdomeric visual photoreceptors. Nonetheless, a key for a complete understanding will be to not only document their presence in but also confirm their functional roles in the visual systems of emerging model systems; to date our attempts with RNAi against *Daphnia Pph13* and *otd2* have not been successful. Fortunately, with the advent of TALEN and CRISPR technology [58], [59] functional studies may no longer be the limiting step. Thus future work will seek to define the ancestral mode of rhabdomeric photoreceptor differentiation among protostomes and in addition determine how the regulatory cascade is modified to generate diversity. For example, it will be interesting to explore how the origin of *Pph13* relates to the diversification of ciliary and rhabdomeric photoreceptors during early metazoan evolution and whether Pph13 or Otd is required for the differentiation of non-visual rhabdomeric photoreceptors, as exemplified by intrinsic photosensitive retinal ganglion cells (ipRGC) [60], [61]; ipRGCs express an r- opsin but do not develop the characteristic membrane folds of a rhabdomere. With respect to Pph13, we have not observed a vertebrate ortholog. While, Arx transcription factors are known to carry out patterning functions in the developing forebrain that could affect the visual system [62], [63] no Arx functions have been reported that relate to the terminal differentiation of photoreceptors. Furthermore, the identification of Otd orthologs as critical components in both rhabdomeric and ciliary photoreceptor cell differentiation [50]–[53] raises the question whether Otd represents the ancestral mechanism for regulating photoreceptor differentiation in both fundamental types of photoreceptors. Overall, these answers will come from comprehending how the differences in phototransduction and morphology between the two fundamental types of photoreceptors are transcriptionally regulated, whether the regulation is conserved within each photoreceptor type, and how transcriptional regulation is modified dependent upon whether the photoreceptor is incorporated into a visual or non-visual system.

## Materials and Methods

### cDNAs, transgenic constructs, and *Drosophila* strains

cDNAs representing *Tribolium Pph13* and *otd2* were constructed from RT-PCR reactions from total RNA isolated from beetle heads. The *otd1* cDNA was a gift from Dr. G. Bucher. cDNAs representing *Daphnia magna Pph13 and otd2* were constructed from RT-PCR reactions from total RNA isolated from whole adults. The *Daphnia magna* cDNA of *otd1* was isolated by screening an embryonic cDNA library [64]. For transgenics, all cDNAs were cloned into pUASTattB vector and integrated into genome position 65B2 (Rainbow Transgenic Flies, Inc.). *Pph13*-GAL4 was generated by inserting the immediate upstream 1.6 kb of genomic DNA extending from the first coding Methionine of the *Pph13* locus into pCHS-GAL4. *Drosophila* strains utilized: *cn bw*, *cn bw Pph13^hzy^* [19], *otd^uvi^* [65], [66], *y w; Sp/Cyo; UAS-Flag-otd/TM2* [46], [47], and *otd^uvi^; otd-GAL4, Pwiz6/Cyo; TM2/TM6B* [46], [47]. For *otd* rescue experiments females of *otd^uvi^; otd-GAL4, Pwiz6/Cyo; TM2/TM6B* were crossed to *w; +; UAS – otd X* transgenic lines and only non *CyO*;*TM6B* male progeny were examined. For *Pph13* rescue experiments *w; cn bw Pph13/CyO; Pph13-GAL4/TM6B* homozygous flies were crossed to *w; cn bw Pph13/CyO; UAS- Pph13 X/TM6B* and only non *CyO; TM6B* progeny were examined.

### Electrophoretic mobility shift assays (EMSAs)

All cDNAs were cloned into pCDNA 3.1(Invitrogen) and EMSAs were performed as described in [19], [67]. Proteins were generated in reticulocyte lysates (Promega). The sequences of probes utilized are listed in Table S1.

### *Tribolium* RNAi injections

To generate RNAi knockdown animals, 1 ug/ul of total probe, DsRNA was injected into *pu11*, *m26, or v^w^* late stage larvae; *pu11* and *m26* contain a 3XP3-GFP and a 3XP3-RFP reporter, respectively [68], [69]. Two independent DsRNAs for each gene were tested and eyes from at least five different subjects were examined to confirm phenotypes. The regions listed for each gene are listed in Table S3. For all the results reported here a mixture of the two DsRNAs for each gene were utilized. The mixtures of DsRNAs contained equal amounts of each individual DsRNA and were used and a final concentration of 1 ug/ul was injected. Dark-reared, newly eclosed (<12 hours old) beetles were collected, scored and utilized for various procedures.

### Reverse transcriptase PCR (RT-PCR)

Total RNA was isolated using Trizol and DNAase treated. First strand synthesis was accomplished with the Superscript III (Invitrogen) kit. Each reaction contained 2 ug of total RNA and oligo-dT as primers. PCR amplification was performed with 1 ul (1/20^th^) of RT reaction and samples were collected at 25 or 30 cycles. All reactions were repeated three times with two independent sets of total RNA. Equal amounts of PCR reactions were analyzed by gel electrophoresis. The list of PCR primers used can be found in Table S4.

### RNA-Seq

For each condition, duplicate sets of total RNA from entire animals (<12 hours old) were isolated. Stranded RNA sequencing libraries were constructed using the TruSeq Stranded mRNA Sample Preparation Kit (Illumina, San Diego, CA) according to manufacturer's instructions. Libraries were quantified using the KAPA SYBR FAST Roche LightCycler 480 2X qPCR Master Mix (Roche, Indianapolis, IN), pooled in equal molar amounts, and sequenced on a HiSeq2000 instrument (Illumina, San Diego, CA) using a 100 bp paired-end run. HISeq read sequences were cleaned using Trimmomatic version 0.30 [70] to remove adapter sequences and perform quality trimming. Trimmomatic was run with the following parameters, “3:30:10 LEADING:3 TRAILING:3SLIDINGWINDOW:4:20 MINLEN:75”. The resulting reads were mapped against the *Tribolium* release 3.0 gene models (http://www.Beetlebase.org/) using TopHat2 version 2.0.9 [71] with the parameters “–b2-very-sensitive–read-edit-dist 2 –max-multihits 100 –library-type fr-firststrand”. Read counting was done for each gene using htseq-count from the HTSeq package version 0.5.4p3 [72] with the “–stranded = reverse” parameter. For *Tribolium*, read counts were normalized across samples using the DESeq package (version 1.12) in R/Bioconductor [73]. DESeq [72] was further used to detect statistically significant differences in expression between two conditions using the binomial test with a .05 adjusted p-value cutoff. The complete data set will be presented and discussed elsewhere but total read data and reads specific to *Tribolium LW* and *UV* opsin are listed in Tables S5 and Tables S6.

### Transmission electron microscopy analysis

*Drosophila* and *Tribolium* heads were prepared as previously described [20], [74]. All samples were from newly emerged adults and for each genotype at least three retinas from three different heads were examined. Samples were observed under transmission electron microscope (TEM), operated at 60 KV and digital images were captured and imported into Adobe Photoshop.

### Antibody production

The *Tribolium* Pph13 polyclonal antibody, 171, was created by injecting rats with a GST-fusion protein representing amino acids 104–220 of the protein. The *Daphnia magna* Otd2 polyclonal antibody was prepared in guinea pigs against a bacterially expressed C-terminal portion of the protein (amino acids 235–395). The *Daphnia magna* Otd1 polyclonal antibody was prepared in rats against a bacterially expressed portion of the protein (amino acids 220–351).

### Whole mount RNA *in situ* hybridization and immunofluorescence

Whole mount RNA *in situ* hybridization in *Tribolium* and *Daphnia* was performed as previously described [42], [75]. The regions of the sense and ant-sense probes are listed in Table S3. The following primary antibodies were used: rabbit anti-Rh6 (1∶2500; Dr. C. Desplan), rat anti- *Tribolium* Pph13 (1∶20), rat anti- *Daphnia* Otd1 (1∶500; Dr. Y. Shiga), guinea pig anti- *Daphnia* Otd2 (1∶500; Dr. Y. Shiga) mouse anti-Rh3 (1∶50; Dr. Steve Britt), mouse anti-Rh5 (1∶50; Dr. Steve Britt) and rabbit anti-Rh6 (1∶1000; Dr. Claude Desplan). For Otd2, in *Daphnia*, signals were amplified with the Tyramide Signal Amplification (TSA) system (PerkinElmer). FITC and Rhodamine conjugated secondary antibodies were utilized (Jackson ImmunoResearch). Immunofluoresence studies were performed as previously described [47], [76]. All *Drosophila* samples were from newly emerged adults and for each genotype at least three retinas from three different heads were examined.

### Sequences

Many of the sequences utilized in this study can be found in Table S7.

## Supporting Information

Figure S1 Phylogenetic tree of Orthodenticle and related homeodomain transcription factors. Maximum likelihood tree estimated in MEGA 5.2.2 [71] from alignment of the homeodomain of the protein sequences listed below or downloaded from HomeoDB: Homeobox Database (http://homeodb.zoo.ox.ac.uk/).
(TIF)

Figure S2 zPhylogenetic tree of Pph13/Aristaless and related homeodomain transcription factors. Maximum likelihood tree estimated in MEGA 5.2.2 [71] from alignment of the homeodomain of the protein sequences listed below or downloaded from HomeoDB: Homeobox Database (http://homeodb.zoo.ox.ac.uk/).
(TIF)

Figure S3 RNA *in situ* hybridizations with sense probes in the developing retina of adult *Tribolium*. A–C. Dissected retinas 30–40 hrs APF of developing *Tribolium castaneum* adult visual system. None of the sense probes for *Pph13* (A), *otd1* (B), *otd2* (C) demonstrate a specific expression pattern in the developing retina. At this developmental time point it is impossible to discern the developing retina field from surrounding tissue without a molecular marker for the photoreceptors.
(TIF)

Figure S4 Phylogenetic tree of r- opsins of *Daphnia pulex*. Phylogeny of 27 *Daphnia pulex* opsin proteins inferred using the Neighbor-Joining method with bootstrap test (500 replicates). Scale bar represents genetic distance as number of amino acid substitutions per site. Tree modified from original data presented in [30]. Red circles indicate r-opsins containing an RCSI site.
(TIF)

Figure S5 RNA in situ hybridizations with sense and antisense probes in the developing retina of *Daphnia magna*. Mixed staged embryos were examined for expression of long wave (LW) A r-opsins (A,B), long wave B r-opsins (C,D), ultra violet (UV) r-opsin (E,F), *otd2* (G,H), and *Pph13* (I,J). Circles represent expression in the putative eye. None of the sense probes demonstrate a specific expression pattern in the developing retina.
(TIF)

Figure S6 Phylogenetic tree of Gβ comparisons. Evolutionary relationships of Gβ proteins of *D. melanogaster*, *T. castaneum* and three *D. pulex* homologs inferred from Neighbor-Joining method with bootstrap test (500 replicates). Scale bar represents genetic distance as number of amino acid substitutions per site. Loci in red indicate Gβ containing an RCSI site. Gβ76C-flybase.org, LOC662674-beetlebase.org.
(TIF)

Figure S7 DNA binding properties of *Daphnia magna* Pph13. Electrophoretic mobility shift assays of *Daphnia magna* Pph13 protein on its endogenous Gβ RCSI site with or without 25 fold excess of cold competitor RCSI sites (Table S1). *Daphnia* Pph13 has the ability to bind to the identified endogenous RCSI sites of the LW r- opsins and like *Tribolium* Pph13 shows differential binding to the RCSI sites identified in the Tribolium LW and UV r-opsins. LW* is a mutated form of LW (Table S1). The arrow indicates the specific mobility shift for *Daphnia magna* Pph13.
(TIF)

Figure S8 Expression of *Daphnia magna* Otd2 in an *otd* mutant background. (A,B) Utilizing the established paradigm of [47], the expression of Otd2 results in the appearance of dead/dying tissue (dark/black patches) “among living tissue *white^+^* (pale yellow) tissue”.
(TIF)

Figure S9 *In vivo* rescue of Rh6 opsin repression in *Drosophila orthodenticle* mutant background. A–E. Rh6 opsin protein expression in adult *Drosophila* retinas. A. Wild-type retina. Rh6 protein accumulates in approximately 70% of the central R8 photoreceptors. B. *otd* mutant. Rh6 expression is detected in numerous photoreceptors in each ommatidium and accumulation is diffuse due to the disruption of rhabdomere morphology. C–G. Rescue of *otd* mutant with: C. *Drosophila* (Dmel) *otd.* D. *Tribolium* (Tcas) *otd1*. E. *Tribolium* (Tcas) *otd2*. F. *Daphnia magna* (Dmag) *otd1*. G. *Daphnia magna otd2*. All Otd orthologs, except Tcas Otd1, are capable of repressing *rh6* transcription and limiting Rh6 expression in a subset of R8 photoreceptors. Insets represent a magnified view of a region of each panel.
(TIF)

Figure S10 *In vivo* rescue of Rh5 opsin expression in *Drosophila orthodenticle* mutation. A–E. Rh5 opsin protein expression in adult *Drosophila* retinas. A. Wild-type retina. Opsin protein accumulates in the rhabdomeres and thus appears as a tube like structure. B. *otd* mutant. The absence of Otd activity results in the absence of *Rh5* transcription and thus no protein is detected. C–G. Rescue of *otd* mutant with: C. *Drosophila* (Dmel) *otd*. D. *Tribolium* (Tcas) *otd1*. E. *Tribolium* (Tcas) *otd2*. F. *Daphnia magna* (Dmag) *otd1*. G. *Daphnia magna otd2*. Only with Tcas *otd2* did we see the expression of Rh5 in the absence of additional *melted* signaling [47].
(TIF)

Figure S11 Knockdown of *Tribolium otd2* affects rhabdomere biogenesis. Transmission electron microscopy analyses of *Tribolium* adult rhabdomeres. (A–C) Three additional samples of phenotypes observed with the knockdown of *otd2*. All samples were from newly emerged adults. Scale bar is 2 um. The removal of *otd2* resulted in rhabdomere degeneration as observed by the large separations between microvilli and extension of microvilli into the cell body.
(TIF)

Table S1 Identification and Sequence of *Daphnia*, *Tribolium* and *Drosophila* RCSI sites. Motif search: Motif search for TAATNNNATTA carried out using perl script “motifsearch.pl” on 1000 bp upstream regions of 27 *Daphnia pulex* opsin genes and confirmed by eye. *Daphnia pulex* genome release 1. Due to gaps in genome coverage, we could not determine whether an RCSI site exists in Daphnia r- opsins: LOPB12, LOPB13, LOPB14, and UNOP1.
(DOCX)

Table S2 Quantification of RNAi injections scored for the absence or presence of 3XP3-RFP.
(DOCX)

Table S3 Probes regions for RNAi and RNA *in situ* hybridizations.
(DOCX)

Table S4 PCR primers for reverse transcriptase-polymerase chain reaction assays.
(DOCX)

Table S5 RNAseq data for Tcas LW and UV opsins.
(DOCX)

Table S6 RNAseq total read counts.
(DOCX)

Table S7 Gene sequences utilized in this study.
(DOCX)

Movie S1 Confocal stack of Otd2 expression (green) in the two visual clusters at 48 hrs after egg deposit (AED) in *Daphnia magna*. The nuclei are counter stained with DAPI.
(MOV)
