# Supplementary figures and images for: Common Transcriptional Mechanisms for Visual Photoreceptor Cell Differentiation among Pancrustaceans
Source: PLoS Genet. 2014 Jul 3;10(7):e1004484. doi: 10.1371/journal.pgen.1004484 (PMC4084641; doi:10.1371/journal.pgen.1004484)

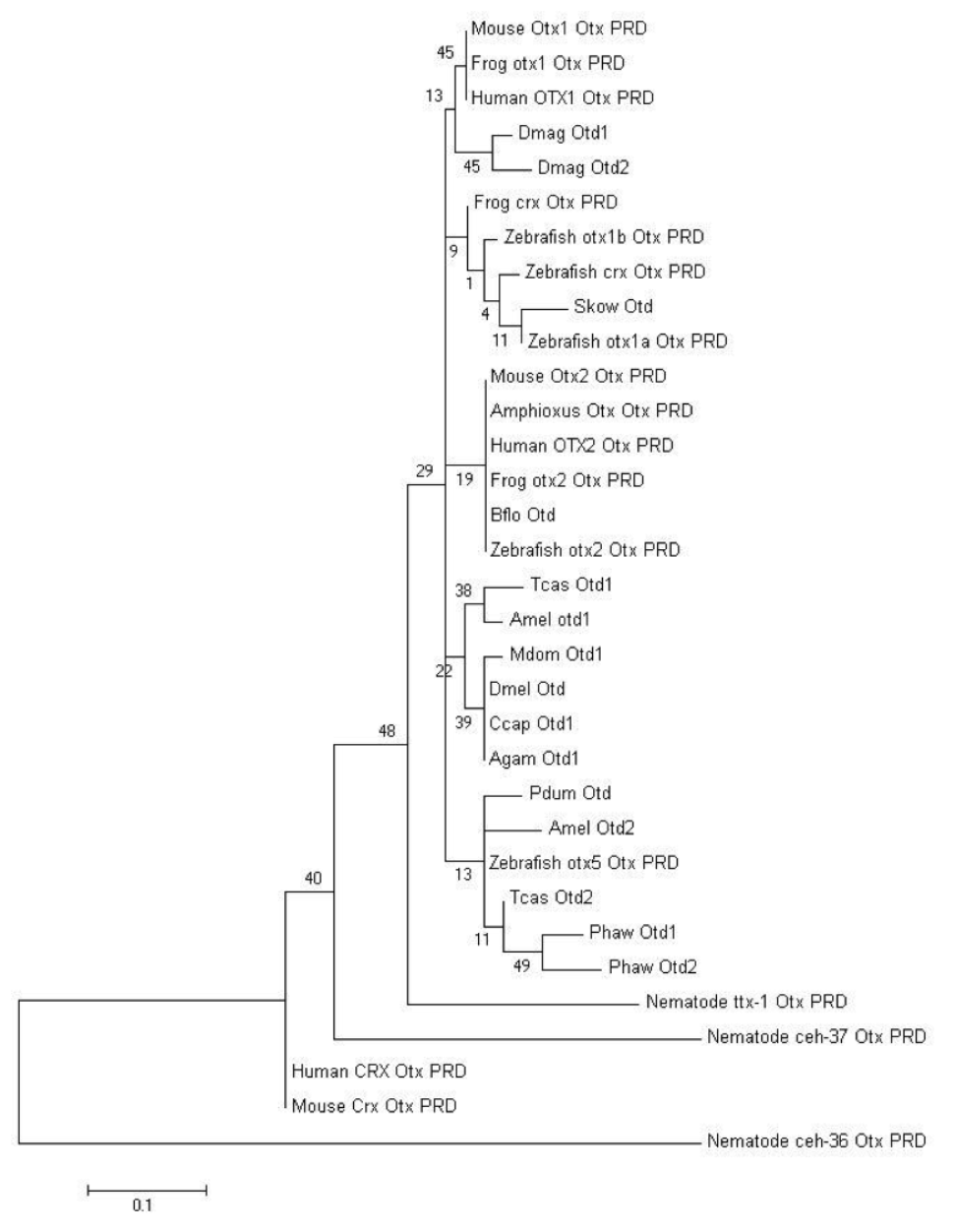

Supplement: Figure S1 — Phylogenetic tree of Orthodenticle and related homeodomain transcription factors. Maximum likelihood tree estimated in MEGA 5.2.2 [71] from alignment of the homeodomain of the protein sequences listed below or downloaded from HomeoDB: Homeobox Database (http://homeodb.zoo.ox.ac.uk/). (TIF) [file pgen.1004484.s001.tif]

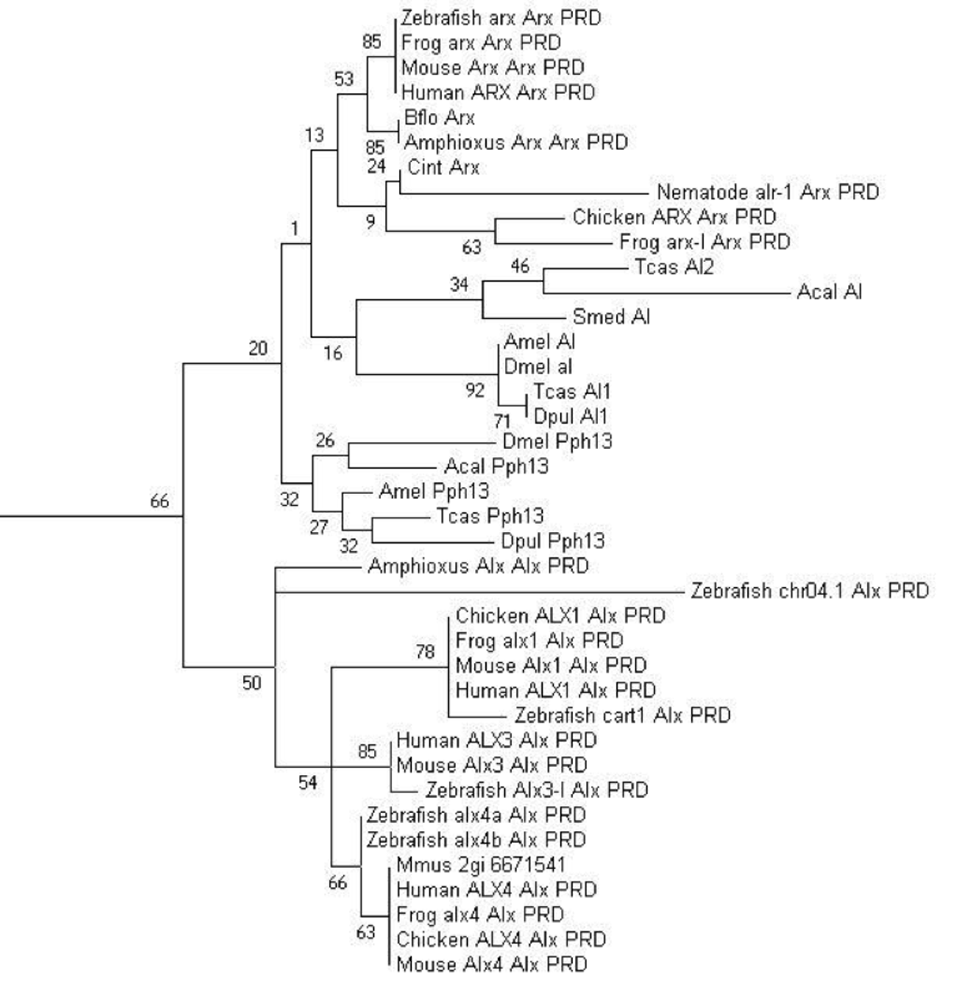

Supplement: Figure S2 — zPhylogenetic tree of Pph13/Aristaless and related homeodomain transcription factors. Maximum likelihood tree estimated in MEGA 5.2.2 [71] from alignment of the homeodomain of the protein sequences listed below or downloaded from HomeoDB: Homeobox Database (http://homeodb.zoo.ox.ac.uk/). (TIF) [file pgen.1004484.s002.tif]

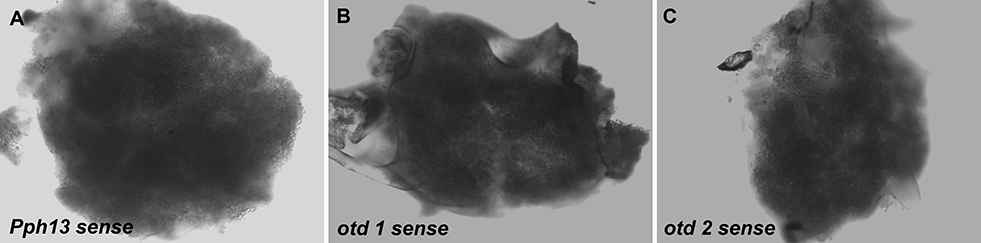

Supplement: Figure S3 — RNA in situ hybridizations with sense probes in the developing retina of adult Tribolium. A–C. Dissected retinas 30–40 hrs APF of developing Tribolium castaneum adult visual system. None of the sense probes for Pph13 (A), otd1 (B), otd2 (C) demonstrate a specific expression pattern in the developing retina. At this developmental time point it is impossible to discern the developing retina field from surrounding tissue without a molecular marker for the photoreceptors. (TIF) [file pgen.1004484.s003.tif]

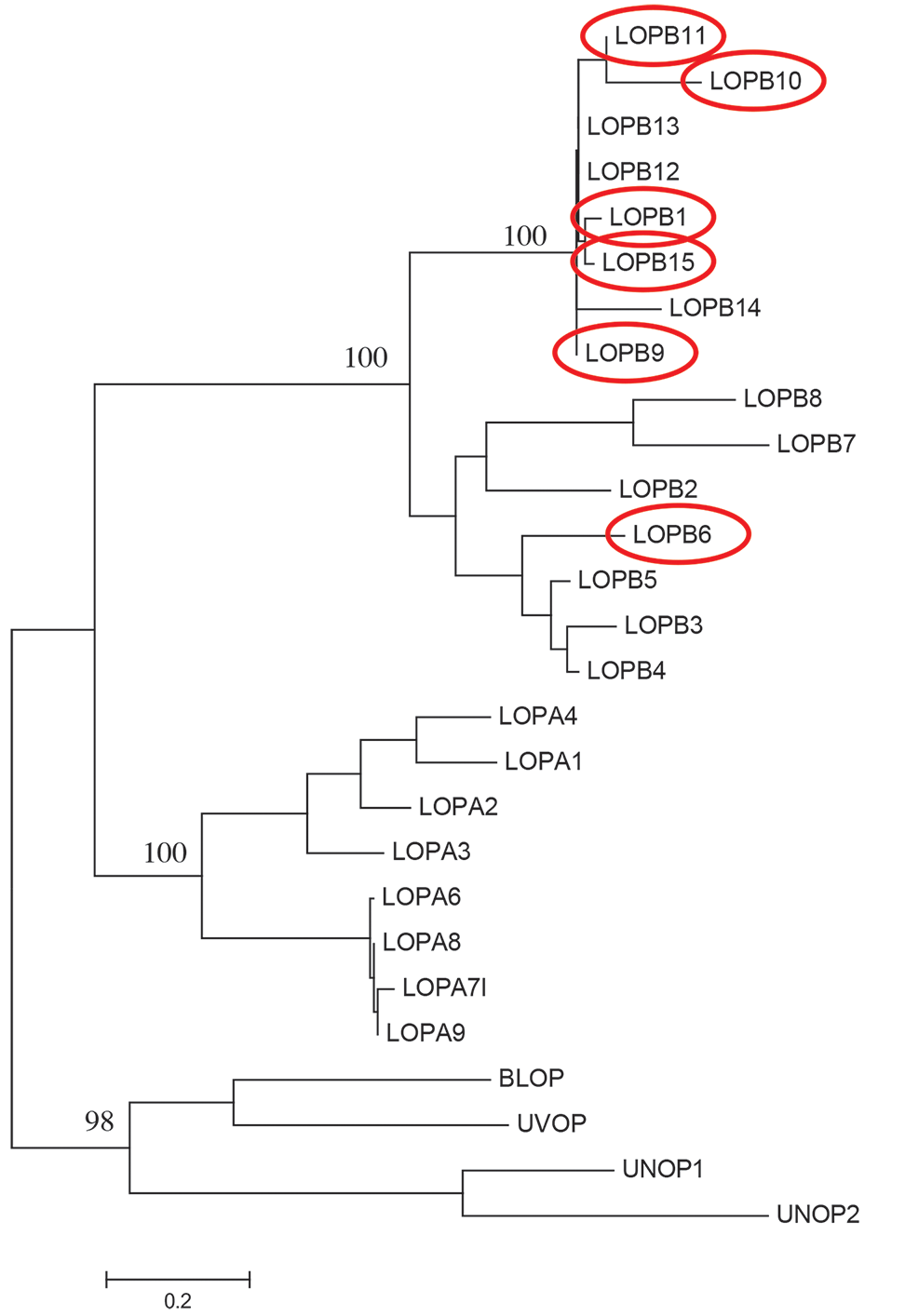

Supplement: Figure S4 — Phylogenetic tree of r- opsins of Daphnia pulex. Phylogeny of 27 Daphnia pulex opsin proteins inferred using the Neighbor-Joining method with bootstrap test (500 replicates). Scale bar represents genetic distance as number of amino acid substitutions per site. Tree modified from original data presented in [30]. Red circles indicate r-opsins containing an RCSI site. (TIF) [file pgen.1004484.s004.tif]

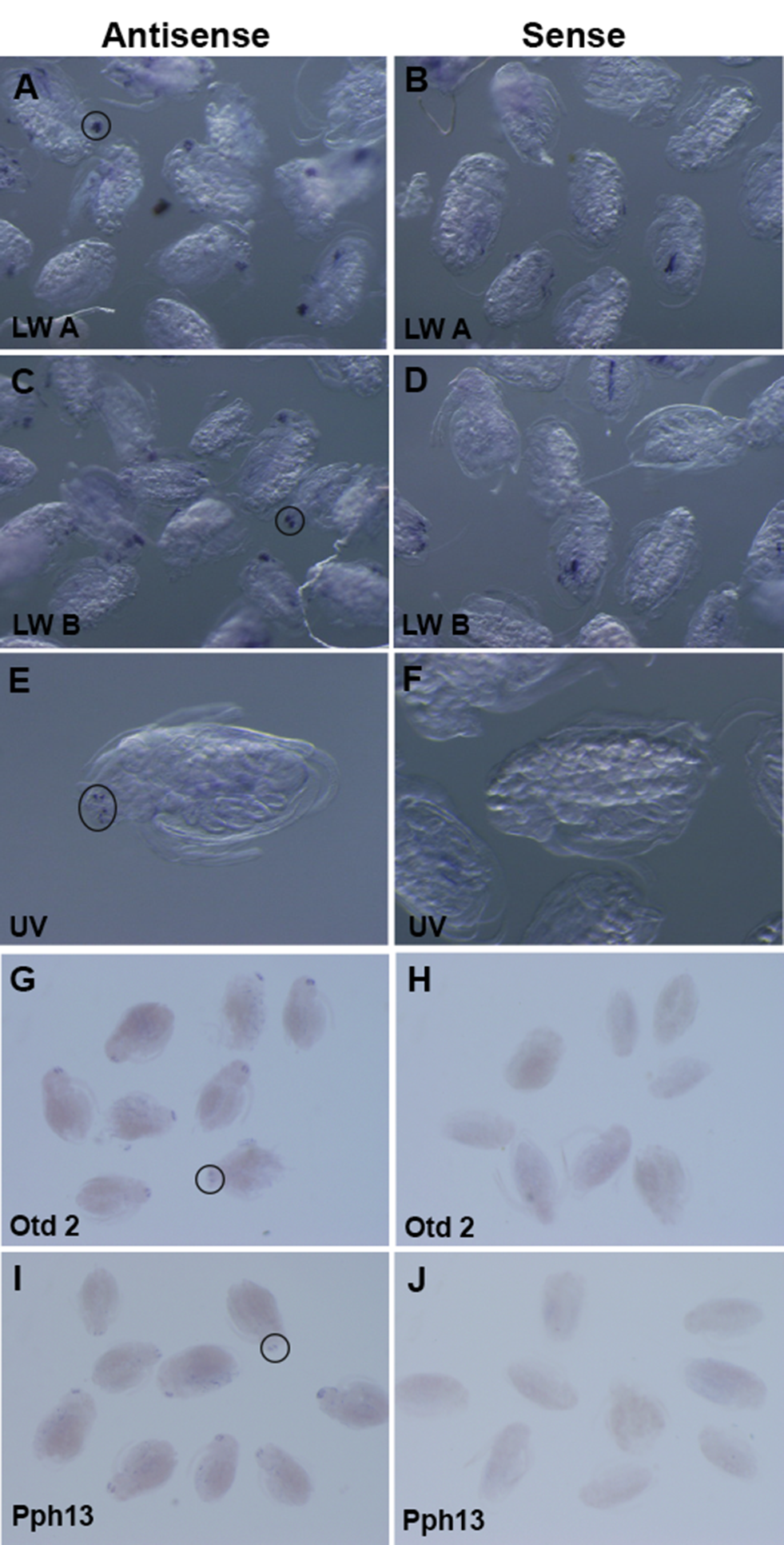

Supplement: Figure S5 — RNA in situ hybridizations with sense and antisense probes in the developing retina of Daphnia magna. Mixed staged embryos were examined for expression of long wave (LW) A r-opsins (A,B), long wave B r-opsins (C,D), ultra violet (UV) r-opsin (E,F), otd2 (G,H), and Pph13 (I,J). Circles represent expression in the putative eye. None of the sense probes demonstrate a specific expression pattern in the developing retina. (TIF) [file pgen.1004484.s005.tif]

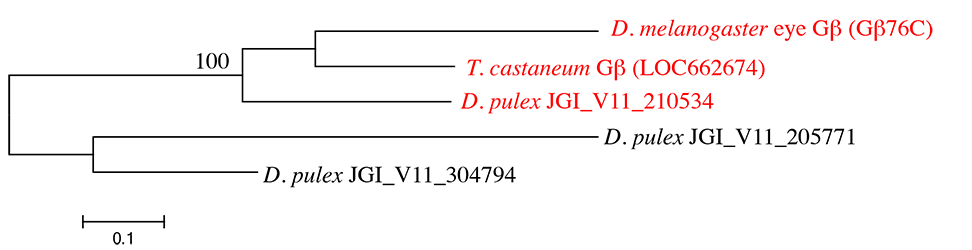

Supplement: Figure S6 — Phylogenetic tree of Gβ comparisons. Evolutionary relationships of Gβ proteins of D. melanogaster, T. castaneum and three D. pulex homologs inferred from Neighbor-Joining method with bootstrap test (500 replicates). Scale bar represents genetic distance as number of amino acid substitutions per site. Loci in red indicate Gβ containing an RCSI site. Gβ76C-flybase.org, LOC662674-beetlebase.org. (TIF) [file pgen.1004484.s006.tif]

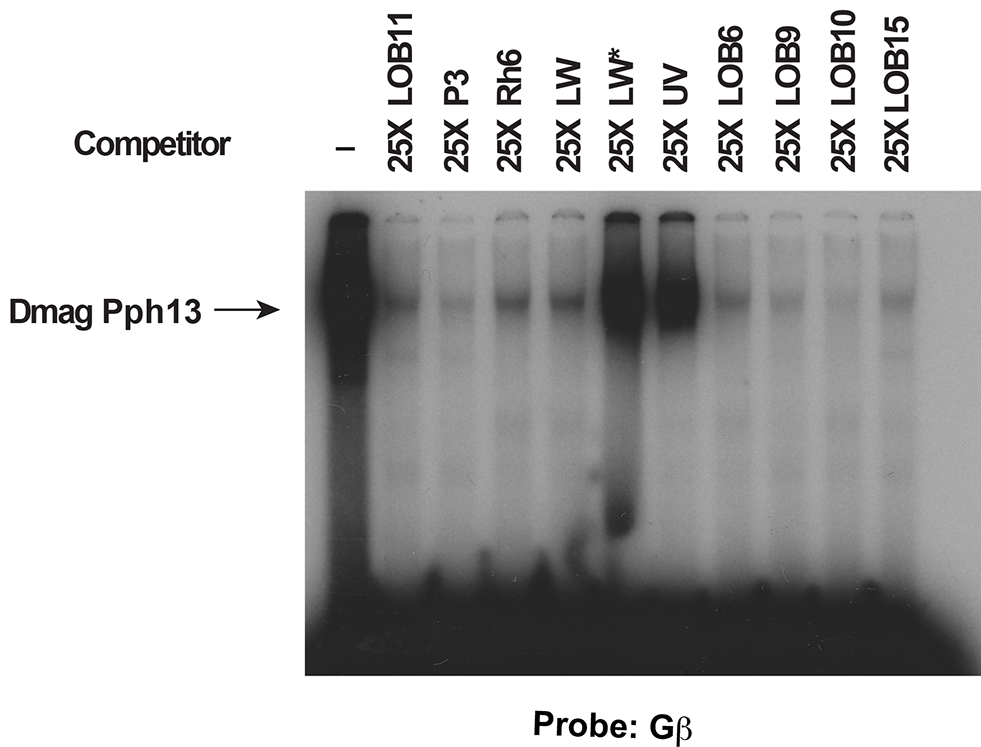

Supplement: Figure S7 — DNA binding properties of Daphnia magna Pph13. Electrophoretic mobility shift assays of Daphnia magna Pph13 protein on its endogenous Gβ RCSI site with or without 25 fold excess of cold competitor RCSI sites (Table S1). Daphnia Pph13 has the ability to bind to the identified endogenous RCSI sites of the LW r- opsins and like Tribolium Pph13 shows differential binding to the RCSI sites identified in the Tribolium LW and UV r-opsins. LW* is a mutated form of LW (Table S1). The arrow indicates the specific mobility shift for Daphnia magna Pph13. (TIF) [file pgen.1004484.s007.tif]

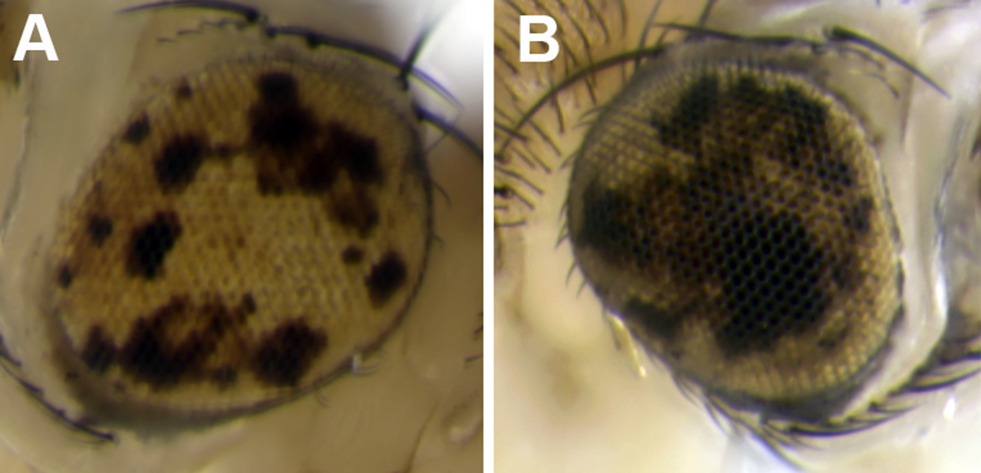

Supplement: Figure S8 — Expression of Daphnia magna Otd2 in an otd mutant background. (A,B) Utilizing the established paradigm of [47], the expression of Otd2 results in the appearance of dead/dying tissue (dark/black patches) “among living tissue white+ (pale yellow) tissue”. (TIF) [file pgen.1004484.s008.tif]

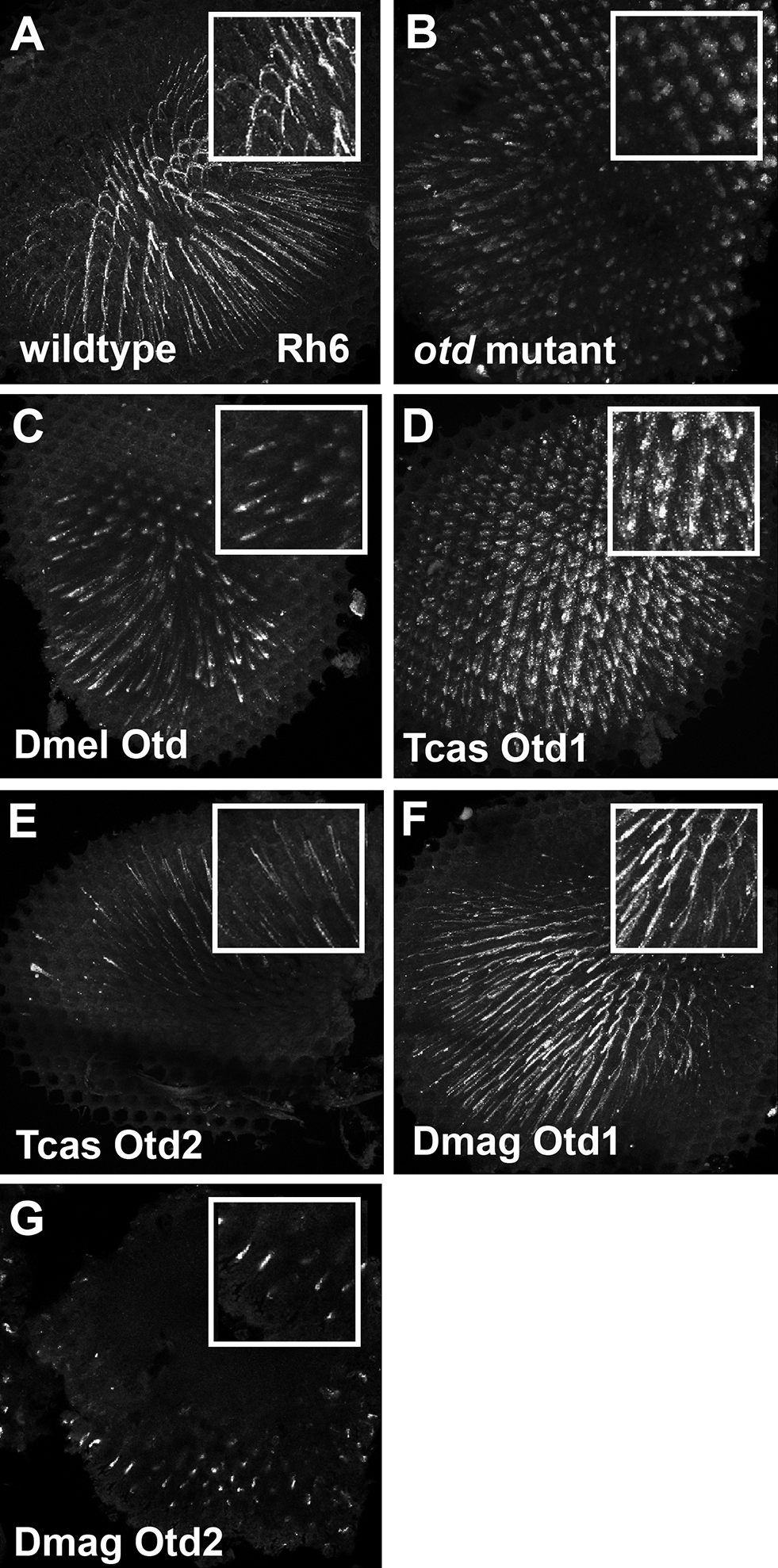

Supplement: Figure S9 — In vivo rescue of Rh6 opsin repression in Drosophila orthodenticle mutant background. A–E. Rh6 opsin protein expression in adult Drosophila retinas. A. Wild-type retina. Rh6 protein accumulates in approximately 70% of the central R8 photoreceptors. B. otd mutant. Rh6 expression is detected in numerous photoreceptors in each ommatidium and accumulation is diffuse due to the disruption of rhabdomere morphology. C–G. Rescue of otd mutant with: C. Drosophila (Dmel) otd. D. Tribolium (Tcas) otd1. E. Tribolium (Tcas) otd2. F. Daphnia magna (Dmag) otd1. G. Daphnia magna otd2. All Otd orthologs, except Tcas Otd1, are capable of repressing rh6 transcription and limiting Rh6 expression in a subset of R8 photoreceptors. Insets represent a magnified view of a region of each panel. (TIF) [file pgen.1004484.s009.tif]

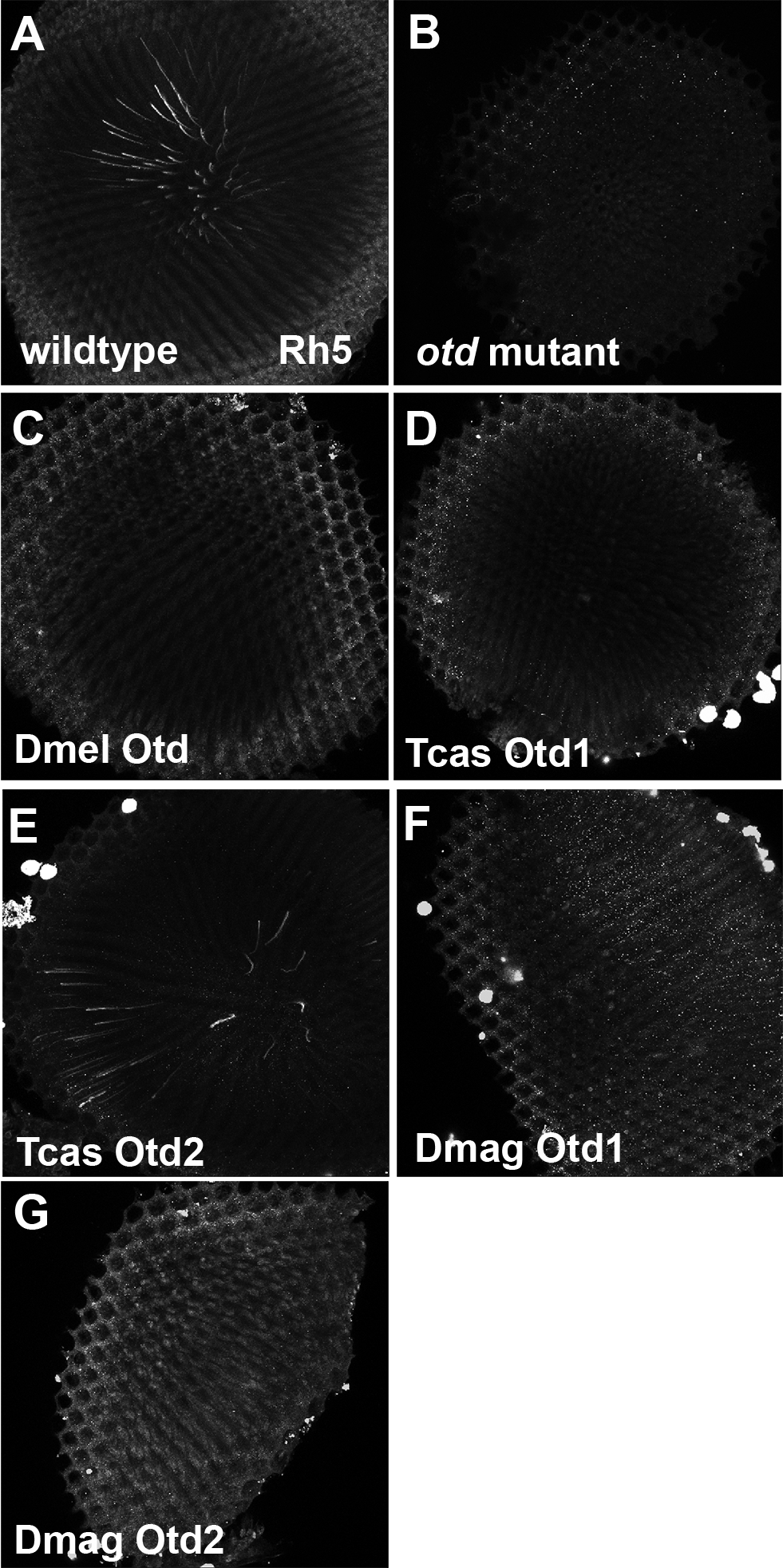

Supplement: Figure S10 — In vivo rescue of Rh5 opsin expression in Drosophila orthodenticle mutation. A–E. Rh5 opsin protein expression in adult Drosophila retinas. A. Wild-type retina. Opsin protein accumulates in the rhabdomeres and thus appears as a tube like structure. B. otd mutant. The absence of Otd activity results in the absence of Rh5 transcription and thus no protein is detected. C–G. Rescue of otd mutant with: C. Drosophila (Dmel) otd. D. Tribolium (Tcas) otd1. E. Tribolium (Tcas) otd2. F. Daphnia magna (Dmag) otd1. G. Daphnia magna otd2. Only with Tcas otd2 did we see the expression of Rh5 in the absence of additional melted signaling [47]. (TIF) [file pgen.1004484.s010.tif]

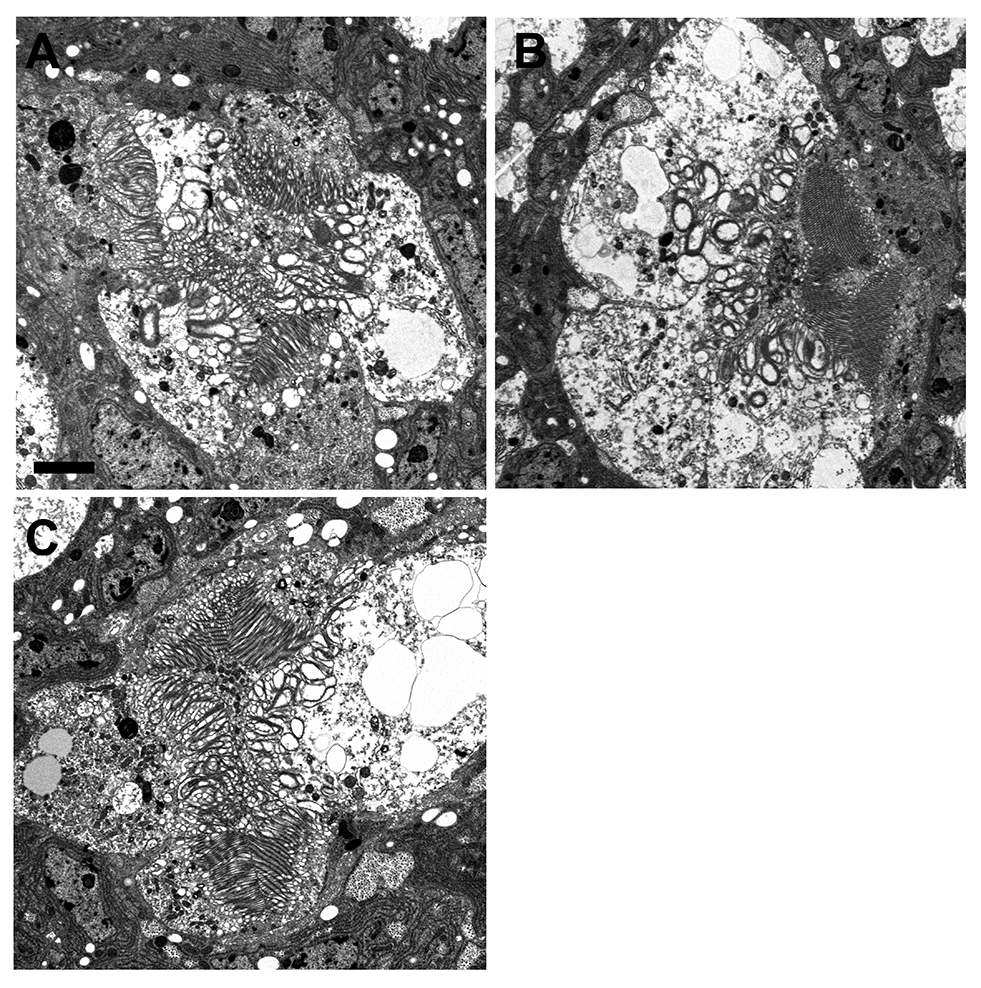

Supplement: Figure S11 — Knockdown of Tribolium otd2 affects rhabdomere biogenesis. Transmission electron microscopy analyses of Tribolium adult rhabdomeres. (A–C) Three additional samples of phenotypes observed with the knockdown of otd2. All samples were from newly emerged adults. Scale bar is 2 um. The removal of otd2 resulted in rhabdomere degeneration as observed by the large separations between microvilli and extension of microvilli into the cell body. (TIF) [file pgen.1004484.s011.tif]
