# Supplementary material for: Common Transcriptional Mechanisms for Visual Photoreceptor Cell Differentiation among Pancrustaceans
Source: PLoS Genet. 2014 Jul 3;10(7):e1004484. doi: 10.1371/journal.pgen.1004484 (PMC4084641; doi:10.1371/journal.pgen.1004484)
Supplement: Table S1 — Identification and Sequence of Daphnia, Tribolium and Drosophila RCSI sites. Motif search: Motif search for TAATNNNATTA carried out using perl script “motifsearch.pl” on 1000 bp upstream regions of 27 Daphnia pulex opsin genes and confirmed by eye. Daphnia pulex genome release 1. Due to gaps in genome coverage, we could not determine whether an RCSI site exists in Daphnia r- opsins: LOPB12, LOPB13, LOPB14, and UNOP1. (DOCX) [file pgen.1004484.s012.docx]

**Table S1:** Identification and Sequence of *Daphnia*, *Tribolium* and *Drosophila* RCSI sites.

| Gene (*Daphnia pulex*) | Sequence (5’-3’) | Distance From ATG (Met) |
| --- | --- | --- |
| LOPB15 (2 sites) | TTCTA **TAAT** TCA **ATTA** TGACC | - 325 bp |
|  | ACATA **ATTA** ATT **TAAT** CAAAG | - 768 bp |
| LOPB11 | TCCTC **TAAT** TAA **ATTA** TGACT | -325 bp |
| LOPB9 | TTCTC **TAAT** TCA **ATTA** TGCCC | -326 bp |
| LOPB6 | ACCTA **TAAT** TCA **ATTA** TAATT | - 447 bp |
| LOPB1 | CCAAG **ATTA** CCT **TAAT** CGATA | -582 bp |
| LOB10 | TCCTC **TAAT** TAA **ATTA** TGACT | - 325 bp |
|  |  |  |
| Gbeta (JGI_V11_210534) | GTTAC **TAAT** TGA **ATTA** GGAGC | - 196 bp |
|  |  |  |

| Gene (*Tribolium casteneum*) |  |  |
| --- | --- | --- |
| LW opsin | GAAGA **TAAT** GTA **ATTA** GGTAG | - 238 bp |
| Gbeta (LOC662674 ) | AAAAC **TAAT** TAG **ATTA** CATGC | - 69 bp |
|  |  |  |
| LW mutant | GAAGA TGAC GTA ATCG GGTAG | Not Applicable |
|  |  |  |
| UV opsin | CGATT **TAAT** AAT **ATTA** AAACC | - 204 bp |

| Gene (*Drosophila melanogaster*) |  |  |
| --- | --- | --- |
| RH6 | TGCCC **TAAT** CCA **ATTA** GCCGG | -205 bp |
| Gbeta76C (2 overlapping sites) | GGC **TAAT** CCA **ATTA** GCTAA | -195 bp |
|  | TAGCT **TAAT** TGC **ATTA** CCG |  |
|  |  |  |
| P3 | TGGCC **TAAT** TGA **ATTA** CAAGA | Not Applicable |
