# Supplementary material for: Common Transcriptional Mechanisms for Visual Photoreceptor Cell Differentiation among Pancrustaceans
Source: PLoS Genet. 2014 Jul 3;10(7):e1004484. doi: 10.1371/journal.pgen.1004484 (PMC4084641; doi:10.1371/journal.pgen.1004484)
Supplement: Table S2 — Quantification of RNAi injections scored for the absence or presence of 3XP3-RFP. (DOCX) [file pgen.1004484.s013.docx]

**Table S2**: Quantification of RNAi injections scored for the absence or presence of 3XP3-RFP.

| RNAi probe | # of adults with RFP | # of adults without RFP |
| --- | --- | --- |
| Mock | 38 | 0 |
| Pph13 | 0 | 30 |
| Otd1 | 26 | 0 |
| Otd2 | 46 | 0 |
| Otd1 and Otd2 | 24 | 0 |
