# Supplementary material for: Common Transcriptional Mechanisms for Visual Photoreceptor Cell Differentiation among Pancrustaceans
Source: PLoS Genet. 2014 Jul 3;10(7):e1004484. doi: 10.1371/journal.pgen.1004484 (PMC4084641; doi:10.1371/journal.pgen.1004484)
Supplement: Table S3 — Probes regions for RNAi and RNA in situ hybridizations. (DOCX) [file pgen.1004484.s014.docx]

**Table S3**: Probes regions for RNAi and RNA *in situ* hybridizations.

| Gene | RNAi Targeted Region |
| --- | --- |
| Tcas *Pph13* | 1-103 aa |
|  | 104-220 aa |
|  |  |
| Tcas *orthodneticle 1* | 1-168 aa |
|  | 233-411 aa |
|  |  |
| Tcas *orthodenticle 2* | 126-202 aa |
|  | 204-300 aa |
|  |  |

| Gene | RNA *In Situ* Hybridization Probe Region |
| --- | --- |
| Tcas *Pph13* | 102-220 aa |
| Tcas *orthodenticle 1* | 191-371 aa |
| Tcas *orthodenticle 2* | 135-300 aa |
| Dmag *Pph13* | 209-569 aa |
| Dmag *UV r- opsin* | 135-378 aa |
| Dmag *LW A-1 r- opsin* | 118-274 aa |
| Dmag *LW A-2 r- opsin* | 147-375 aa |
| Dmag *LW A-3 r- opsin* | 164-382 aa |
| Dmag *LW B-1 r- opsin* | 191-408 aa |
| Dmag *LW B-2 r- opsin* | 170-377 aa |
| Dmag *LW B-4 r- opsin* | 121-366 aa |
