# Supplementary material for: Common Transcriptional Mechanisms for Visual Photoreceptor Cell Differentiation among Pancrustaceans
Source: PLoS Genet. 2014 Jul 3;10(7):e1004484. doi: 10.1371/journal.pgen.1004484 (PMC4084641; doi:10.1371/journal.pgen.1004484)
Supplement: Table S4 — PCR primers for reverse transcriptase-polymerase chain reaction assays. (DOCX) [file pgen.1004484.s015.docx]

**Table S4**: PCR primers for reverse transcriptase-polymerase chain reaction assays.

| Gene | 5’ | 3’ |
| --- | --- | --- |
| Tcas *LW* opsin | 5GGCGGCAACCTCACCGTCGTGGACAAAGTC3 | 5CTGCTTCGGAGGATCGCAAGGACGCAACGT3 |
| Tcas *UV* opsin | 5GCTCGTATACCCCGAACCAGAAGCCTCCAT3 | 5CCACAAACTTGCATGCACAAGCCGGGACCA3 |
| Tcas *RpS3* | 5CTTCCGCTCGCCACACGTCAAACCTTTTCC3 | 5CCACAACAGAGACATTGTCAGGCAGAGGCT3 |
