# Supplementary material for: Common Transcriptional Mechanisms for Visual Photoreceptor Cell Differentiation among Pancrustaceans
Source: PLoS Genet. 2014 Jul 3;10(7):e1004484. doi: 10.1371/journal.pgen.1004484 (PMC4084641; doi:10.1371/journal.pgen.1004484)
Supplement: Table S5 — RNAseq data for Tcas LW and UV opsins. (DOCX) [file pgen.1004484.s016.docx]

**Table S5:** RNAseq data for Tcas LW and UV opsins

|  | **TC013765 (LW opsin)** | | | |
| --- | --- | --- | --- | --- |
|  | **Read Counts** | **Normalized Read Counts** | **Log2 Fold Change** | **Adj. P value** |
| wt v Pph13 | 464 v 31 | 160.7313 v 16.6172 | -3.27390368 | 3.97E-05 |
| wt v OTD1 | 464 v 359 | 165.7437 v 235.5013 | 0.506780324 | 1 |
| wt v OTD2 | 464 v 259 | 175.5685 v 147.7318 | -0.249053818 | 1 |
| wt v OTD1,2 | 464 v 202 | 192.7570 v 125.5089 | -0.618992844 | 1 |
|  |  |  |  |  |
|  |  |  |  |  |
|  |  |  |  |  |
|  | **TC000118 (UV opsin)** | | | |
|  | **Read Counts** | **Normalized Read Counts** | **Log2 Fold Change** | **Adj. P value** |
| wt v Pph13 | 106 v 48 | 36.5696 v 35.9328 | -0.025344543 | 1 |
| wt v OTD1 | 106 v 104 | 37.7092 v 68.3276 | 0.857554265 | 1 |
| wt v OTD2 | 106 v 2 | 39.9451 v 0.7501 | -5.734697621 | 0.002374628 |
| wt v OTD1,2 | 106 v 3 | 43.8548 v 1.6334 | -4.746817812 | 0.002155006 |
