# Supplementary material for: Common Transcriptional Mechanisms for Visual Photoreceptor Cell Differentiation among Pancrustaceans
Source: PLoS Genet. 2014 Jul 3;10(7):e1004484. doi: 10.1371/journal.pgen.1004484 (PMC4084641; doi:10.1371/journal.pgen.1004484)
Supplement: Table S6 — RNAseq total read counts. (DOCX) [file pgen.1004484.s017.docx]

**Table S6:** RNAseq total read counts

| **Condition** | **Unprocessed Reads** | **Trimmed Reads** | **Mapped Reads** | **Gene-Mapped Reads** |
| --- | --- | --- | --- | --- |
| Tcas wildtype | 8,191,485.00 | 5,721,367.00 | 4,449,263.00 | 3,378,997.00 |
| Tcas Pph13 RNAi | 5,057,921.00 | 3,437,511.00 | 2,579,037.00 | 2,039,621.00 |
| Tcas OTD1 RNAi | 6,826,414.00 | 4,575,196.00 | 3,758,396.00 | 2,974,089.00 |
| Tcas OTD2 RNAi | 11,839,906.00 | 7,534,797.00 | 6,272,809.00 | 5,030,033.00 |
| Tcas OTD1,2 RNAi | 14,240,764.00 | 8,697,425.00 | 7,136,000.00 | 5,712,365.00 |
| Tcas wildtype | 14,663,853.00 | 9,455,861.00 | 7,747,811.00 | 6,245,808.00 |
| Tcas Pph13 RNAi | 9,011,353.00 | 5,945,355.00 | 4,745,711.00 | 3,814,482.00 |
| Tcas OTD1 RNAi | 5,030,360.00 | 3,476,317.00 | 2,795,875.00 | 2,298,413.00 |
| Tcas OTD2 RNAi | 4,820,850.00 | 3,100,376.00 | 2,311,925.00 | 1,778,404.00 |
| Tcas OTD1,2 RNAi | 5,404,625.00 | 3,543,959.00 | 2,783,045.00 | 2,185,119.00 |
|  |  |  |  |  |
| **Totals** | 85,087,531.00 | 55,488,164.00 | 44,579,872.00 | 35,457,331.00 |
