# Supplementary material for: Common Transcriptional Mechanisms for Visual Photoreceptor Cell Differentiation among Pancrustaceans
Source: PLoS Genet. 2014 Jul 3;10(7):e1004484. doi: 10.1371/journal.pgen.1004484 (PMC4084641; doi:10.1371/journal.pgen.1004484)
Supplement: Table S7 — Gene sequences utilized in this study. (DOCX) [file pgen.1004484.s018.docx]

**Table S7: Gene sequences utilized in this study.**

**Otd Sequences:**

>Dmel_Otd

MAAGFLKSGDLGPHPHSYGGPHPHHSVPHGPLPPGMPMPSLGPFGLPHGLEAVGFSQGMWGDLCYPGVNTRKQRRERTTFTRAQLDVLEALFGKTRYPDIFMREEVALKINLPESRVQVWFKNRRAKCRQQLQQQQQSNSLSSSKNASGGGSGNSCSSSSANSRSNSNNNGSSSNNNTQSSGGNNSNKSSQKQGNSQSSQQGGGSSGGNNSNNNSAAAAASAAAAVAAAQSIKTHHSSFLSAAAAAASGGTNQSANNNSNNNNQGNSTPNSSSSGGGGGSQAGGHLSAAAAAAALNVTAAHQNSSPLLPTPATSVSPVSIVCKKEHLSGGYGSSVGGGGGGGGASSGGLNLGVGVGVGVGVGVGVSQDLLRSPYDQLKDAGGDIGAGVHHHHSIYGSAAGSNPRLLQPGGNITPMDSSSSITTPSPPITPMSPQSAAAAAHAAQSAQSAHHSAAHSAAYMSNHDSYNFWHNQYQQYPNNYAQAPSYYSQMEYFSNQNQVNYNMGHSGYTASNFGLSPSPSFTGTVSAQAFSQNSLDYMSPQDKYANMV

>gi|525343545|ref|NP_001266352.1| homeotic protein ocelliless-like [Ceratitis capitata]

MAAGLLKSGDFGHHPHSYGGPHPHHSVPHGPLPPGMPMTSLGPFGIPHGLDAVGFSQGMWGVNTRKQRRERTTFTRAQLDVLEALFGKTRYPDIFMREEVALKINLPESRVQVWFKNRRAKCRQQLQQQQQTNSLNSSKNVSGSSSCSSSRNTSNSGNSNNNSSSSNANKSNSHSSHSTSGGSGGNKSNNSSSSGANANNPSGSSAAAAAAAAAVAQSIKSHHSSFLNSSATSGSALTPTSGSAVSSHAHSTHPHHMTGSGINSMASGPSVATVNSLNASVAAHQNSSPLLPTPATSVSPVSIVCKKEHIGAGYAGGGPLGGANDGSMRANYDALKHEGGDISSSVAHHH

SIYGTAASTNPRLVQPGGNITPMDSSSSITTPSPPITPMSPQSAAAAAHAAQNAQSAHHSAAHSAYMPNHESYNFWHNQYNQYPNNYAQAPSYYSQMEYFSNQNQVNYNMGHTGYSASNFGLSPSPSFTGTVSAQAFSQNSLDYMTPQDKYVNMV

>gi|557779867|ref|XP_005189558.1| PREDICTED: homeotic protein ocelliless-like [Musca domestica]

MAAGFLKSGDLGPHPHSYGGPHPHHSVPHGPLPPGMPMPSLGPFGLPHGLEAVGFSQGMWGVNTRKQRRERTTFTRAQLDVLESLFGKTRYPDIFMREEVALKINLPESRVQVWFKNRRAKCRQQLQQQQQSNSLSNSKSVNSASGSCSSSSSSRNASNSSGSNSNSTNSSSKSNNHHSSSSSSSAGGSHSHNNNGNKNSANSNNPNGPSGGAGSSSAAAAAAAAVAQSIKSHHSSFLNSSSSATSAVTPTSSAGNSHLHHMNANSHAAANASAAGLNTSGGGGGGAGHHNSSPLLPTPATSVSPVSIVCKKEHIGSSGYPVSNPDILRAASYDTLKESGGDIGSSGHHHSIYGTAASANPRLLQPGGNITPMDSNSSITTPSPPITPMSPQSAAAAHAAQSAQSAHHSAAHSAAYMSNHDSYNFWHNQYQQYPNNYAQAPSYYSQMEYFSNQNQVNYNMGHSGYSAASNFGLSPSPSFTGTVSAQAFSQNSLDYMTPQDKYVNMV

>gi|347963388|ref|XP_310918.5| AGAP000215-PA [Anopheles gambiae str. PEST]

QQHPSTVALHSTEALMAGFLKSSDLGPHPHGYGSAHHPHHAHPHGPLPPGMPMTSLAPFGLPHGLDAVGFPQGMWGVNPRKQRRERTTFTRAQLDVLEALFGKTRYPDIFMREEVALKINLPESRVQVSVGEVWFKNRRAKCRQQLQHQSSSNLNSSKSSGGGSTGSARSGQSGGNSGPSGSQSGSSGGTGSNNGSHGGTKSSIVNANNKPTIKHTPMNNSHNGSGPGTGSNGGGGGGGAGSQSNPAGPSHNGPLGLSHSNASPILPITPSTSVSPPINVICKKEQPSSYHHGPAGGGGGGGGNEPSGMKPTGYDLLKDSELNLAHHSSSAYVNINTRLGQSLSSVGGGVGGGGGGVGSGGTSHNPLSYVPNHETYNFWHNQYQQYPNNYNTPSYYSQMDYFQNQNQGNYNMGHTGYTTSANFGLASTSALAGPMGTQTFSPNGLDYMNPQDKYVNMV

>Tcas_Otd1

MWPPEAVHSLERFCTYGNVNYRQRRSQPLKISCLKTTPPPEEDNRGCAASVGEGSFYLPMNMQGFVKQQTAPHGPPYAPHPSLSSGLGGGLSGMPMPALGFGLGHPLESVPFPQVYSYFAGVNPRKQRRERTTFTRAQLDLLEGLFAKTRYPDIFMREEVAVKINLPESRVQVWFKNRRAKCRQQLQQQQNKSASRTTTSPTKVKASKASPAAAPRSVATPTGIPTPSTSASPPTVNIKKESPQMQSYRPTGNITPHGSNTSSLITTPSPSASPLAYQHEYNSFNWTANGHGHNTSSHNYYAQNYAPTYYGQMQPDYLNSQTTQNHMQAMNNMAGTYQMTGYSAMGMAAPHHQNFGPRHPPDCSMEFANMA

>Amel_otd1

MACDTGRQVSTILSFTVNTCPQVEDYEGYNSCARNGNVSVSQEPATKVSESSAKLSSQPSQNDHREENSWWTAEEGGGQEIRERDKGAAVAGELACLAGYMAGYLKAAGATCAPTGSPQYHPHSHPAMGVATHPHPHSHPHPGAPHPGLPSPFALATHGHPHPHPLEHGLAAFPQGMNQRKQRRERTTFTRAQLDVLEGLFTKTRYPDIFMREEVALKINLPESRVQVWFKNRRAKCRQQLQQQQQQQQQQQQQQASPSKGSPRGSGQAQSNSSNGNKMSTSSTATPSRRSSPVSPPRGKEAPGPNSPLLSTTSTYPRLGVTPTGGSGANSALTTPSPPLTPGSSQLPPSSYPPPMNQIHHGEYGFTWSSASPGSVNPSQCYAGQTYNAYQNPYTSGDYYQTQISHMHHSPQGNYHHPQYHHNMALTSSMSHLTSHHLNASGPNEMTASPSESDGYILPDQKYQTMV

>Dmag_Otd1

MAYLAKGNPYAMNMGVGMGPVGLHHHHSQGGLGHVGGGGMPGLAGLAMPPSAMDPLHSAVGFPPGMDRPWSNGAGRKQRRERTTFTRGQLDVLESLFAKTRYPDIFMREEVALKINLPESRVQVWFKNRRAKCRQQSQQQQQQQQQQQSTGGSSGSSKSTGTPRPKKIKNSSPGSPVSSRQQQQQQQQQQQSSIINSTPSQAGLSLQQQQQQIQQQQQQHQTKMHRDCSPPSSLPLHSPHTPSSSVTPTPTPPPLGAGGLTGGYGTMVAGMVDVSTHASTGGGVGGVGGGYGNGGNTNFWGGHDFHVPSPQRAGSYGLATGPKMTGSSSSVGTPMSGSMGSFSSPSSMHGMSPMGMMPTGYPSPNPSPAGCYGVATAGGYAAAAAAAANAAPYYSNVGMDYHHHHHTTHSMASHHHSMGSYGQMSSYPGSAQSAYASASAAAQAAVQANHHHHHGGVIGNSRPALPSTVGVQQQQQQQQQQQDCLDYEAKFQVL

>Parhyale Otd1

QRRERTTFTRAQLDVLETLFGKTRYPDIFMREEVAIKINLPESRVQVWFKNRRAKCRQQNTN

>Tcas_Otd2

MWSNSLTAGCNPDSELFPGFGSTCGGSSSSMAYLKSAPYPVPGLGLHGLPVDSLHSSMAGYPAGNQRKQRRERTTFTRAQLDVLEALFGKTRYPDIFMREEVALKINLPESRVQVWFKNRRAKCRQQQKQHNQQQSVEKSSKLKNKSAPILTKTSPTPVSINNNTSTSSSASSPSVTAAPHLRDSPNYIKPQLHVSTGSTSSPTIASATYTNSANSSIWSPASIDSFTLEQHRSWCSSSQPVLSTTNSTTNCYNNYPYYSNMDYLSSSTMSHSQFGNGLETSWGKSRDESSWFYNSGWERK

>Amel_otd2

MWPNSLGGTSGCSGGAGATDLGGLAASTTSASDLFGPAAFGASAAGPYGALKTNPYVSALGMPPIEALHSTIGYPGCTPAGNPRKQRRERTTFTRAQLDVLEGLFSKTKYPDIFMREEVAMKINLPESRVQVWFKNRRAKCRQQQKQQQQQQDKAPRSKKPSGNPSNNPPSGKSPSIATTPTPAAAVPATTPLSGGTGGSAASSPALLRDSPQYKPAGSATSLLLAASTTPPSLGGTVYSSGGSSSSIWSPAVTESGGGFPSDHQRLAWTTTTSQQQCYQNYSSYYTNMDYLSPATHQLNVVDGGGSSLDNTWSKTRDESASSWFYNSAGWGDRK

>Dmag_Otd2

MAMGMSMTSDPQHHPMGSMNGLSYSHGGGGRKQRRERTTFTRGQLDVLESLFAKTRYPDIFMREEVALKISLPESRVQVWFKNRRAKCRQQQKQQQQPHNAEGGNSKGEKRIRKRGPATTTISSTTSDPSTKPSGNVAAMHPVSSVVPPESSGGPSLSSVTMAGSAAACSSMTTEGDSSLISSPYGNLAMALSPIPANMMCNSNSPPLIQQQQLIQQHPSFGSATSSTSSSSNSNATNYAAASYQYHQQSINHFHQHPSSFATPTTNSSSLSGFFPQYTTANTPTVDMMLSSPTIVAAVSNINSSGTAAGMNCVASGGNNGANQASVSSSFWHSPPPMIDYGSSPSMLLPSSSSTQSSTSYYSSLDYSLYAPSLHLPIAKSSGVDEVDQLIHRDACTKNSSPVETDDDQSWAKYTNFQIL

Parhyale hawaiensis otd2 - homeodomain - RRERTTYTRAQLDILETLFGKTRYPDIFMREEVAIKINLPESRIQVWFKNRRAKCRQ

>gi|11877290|emb|CAC19028.1| homeobox transcription factor [Platynereis dumerilii]

MAGMTAYPSGPVGPTSGAVPTSKSSMPYAVNGISLTGPSVDLVHPAMNYQDTRADLEVIQYEAANPRKQRRERTTFTRAQLDVLESLFQKTRYPDIFMREEVALKINLPESRVQVWFKNRRAKCRQQQKAQDGRNPASRPKKSKSPTPGDVSPVNNNTSSDSSYKTVAPVTSTPTPTVNGMGSAGSGGGSGGSQTNSPSIWSPAS

>gi|2828716|gb|AAC00193.1| amphioxus Otx transcription factor [Branchiostoma floridae]

MAYMKSPYGMNGLSLSNPSIDLMTHHHHPGVGVSQYYNPTSAYTVTGQCPPPPRKQRRERTTFTRAQLDVLEALFAKTRYPDIFMREEVALKINLPESRVQVWFKNRRAKCRQQAGQAKPRPKKKSASPPPASTEEQAPTSESPSCSDTSVSSTPVPVAVTIGNTNASPTAAVSTSSIWSPASAPSVTSDLGSCTSTVTSAPSCMQGSSYNHAYNHGGYTAHAQTYSPAPYPPTTYSTSYFGGMADCSSYLSPMAPTQPLPPVTTLNQMSSANMSAHSMSHTPQLSPSSMGHGSPLNMSTQPDCVDYTKHDQTSAWHKFQVL

>gi|259013293|ref|NP_001158360.1| orthodenticle [Saccoglossus kowalevskii]

MMSYPLPHMKPGAPYSVNGISLASPNAVELNQHPAMYPGTIIHGRAPLPLHYFCNPRKQRRERTTFTRAQLDILESLFGKTRYPDIFMREEVALKINLPESRVQVWFKNRRAKCRQQEQQKQNGGQNVNKSRPTHKKGSPPPRDTPPTTTTSMSAMSTMSSMTAPYKSPVVSNGNGIWSPASIPPPVNDHLSSSSSCMQRSTYPMTNAQSPGYSQNYQPSSYFGNIDCSSYLPPMQLPGTTLNQMSSSSMAGSHMSSAPSQLQPSHHMNPATPTGSMGSSMGMHSSLSADSCLESKDHGSSWKFQVL

**Pph13/Al/Arx sequences:**

>gi|75029521|sp|Q9XZU0|Q9XZU0_DROME CG2819-PA (FI01102p) (Munster protein)

MDKGTMKRKQRRYRTTFNTLQLQELERAFQRTHYPDVFFREELAVRIDLTEARVQVWFQNRRAKWRKQEKIGGLGGDYKEGALDLDVSYDDSAVLGQLDSALGGGGTLLPDTPPQSSNSLDNELKASYGTGAMSPSRLSPNIFLNLNIDHLGLERGGSGLSMEWSTYPPQTQAQTHPQMDSDNQLQQHPPQQHASDPIHAGSSSHHQQQQQQHQQEQHNPQLHPGLEFAASLSLDMTDGSSAYDEMKFLSVDVDQFTIDSFKADCILSMEQSQMQAYGGHSQLVGSSNELCLDGIGMSSFGMEEGEPKSPPSLLVLDKSLPSLSIGVEGIADLVEQLHHHQHEGGPVGGG

VITGDVM

>8tr|D6WYT5|D6WYT5_TRICA Munster OS=Tribolium castaneum GN=Mu PE=3 SV=1

MEEKASPGPSYAINTFLNNLDFEKEDRQDYHNYAEDDANNASKRKQRRYRTTFSNYQLEELERAFHKTHYPDVFFREELALRIDLTEARVQVWFQNRRAKWRKQEKSVKNAQNINIPSACSTPLESPLLNFPPEQSPTNLFLGLEWPSIVPQVPYQSVDNINETVLIGDRIAGIHTNLIG

DGILLGEEGNLDLIQNDPEIGIDPDLLTLKPARHNRSEDC

>gi|110762829|ref|XP_001121339.1| PREDICTED: hypothetical protein LOC725502 [Apis mellifera]

MDLKEEDESANENGRQLFLVGAPRDPLVSTGAIVASAGAPDEINSAGDLQSTALAGLIGAQSSSVCFPTKFSYDFSSSPGSEDRQSTGVGKRKQRRYRTTFTNFQLEELERAFQKTHYPDVFFREELALRIQLTEARVQVWFQNRRAKWRKQEKQCKVTTHLTSHLPPSECQEVQQQQQQQQSDHLLLEPPLGSPPPIYLGMEWAGFSPYSNTATASSLVVNSMNKPLESEADDNPLLDPELLQLKTPRS

>Dpul_Pph13

RRYRTTFTNFQLEELEHAFHKTHYPDVFFREELAVKIDLTEARVQVWFQNRRAKFRKHE

>gi|524913030|ref|XP_005111349.1| PREDICTED: homeobox protein aristaless-like 4-like [putative Pph13] [Aplysia californica]

MEGQFSALLTSDDIGFLDSPDATSGFYFPLCGTMEDTGLTRSRFDPPDSLCMAERSGNDGRVSYTISGLLGPNPNQPMPSPPPPPPPPHHLSSQPPVLTSQGPPPPPPPAPPSQQSVDSGFSFNPSHQHSLHGRDQSHDPHARNSDNGNHGYSVGGGVPGGPDPAGDGVRVQRSRDVRDTGDYVNQSFIGSTSRRKQRRYRTTFNSSQLEELERAFQKTHYPDVFFREELALKIGLTEARVQVWFQNRRAKWRKQQKEEQKSRLVTDTGCHALKASQNKPPPQPPPPMSSPACKMTSFGSVPVPGFYFHGNLNVDWTSQMNPSMSSSSLTSFLASKNGQRYQGSMDENSFGGRCSGQTQGDSGSMKTNLHDNQFQLPDAVYTRDARVGSLVALRLKAQEHQEAIKA

>4gi|290262|gb|AAA28840.1| prd-like homeobox protein [al] [Drosophila melanogaster]

MGISEEIKLEELPQEAKLAHPDAVVLVDRAPGSSAASAGAALTVSMSVSGGAPSGASGASGGTNSPVSDGNSDCEADEYAPKRKQRRYRTTFTSFQLEELEKAFSRTHYPDVFTREELAMKIGLTEARIQVWFQNRRAKWRKQEKVGPQSHPYNPYLPGGAATMQTVVGAALPPNPFTHLGFQLRKPFDAQHAANLAAFRYPHLSAAPMIPSGYFNQFQRAPPHMLPHGMAGMYSPSSSFQSLLANMTAVPRGPPLGKPPALLVGSPDLHSPNHMLASPPTSPASGHASQHQQHPTAHPPPPQAPPQMPVGVQPAQLSPQQLVGIALTQQASSLSPTQTSPVALTLSHSPQRQLPPPSHQAPPPPPRAATPPEDRRTSSIAALRLKAREHELKLELLRQNGHGNDVVS

>3gi|167234447|ref|NP_001107838.1| aristaless [Tribolium castaneum]

MDLSTRQRDPQDNAKMRVAKSVFSIRSLVDLGDAADAPDDDAPKEPVASDRNPEVMGVSEEVTPNAGVEPRPPGPDNSDGSDPEPDEFAPKRKQRRYRTTFTSYQLEELEKAFSRTHYPDVFTREELAMKIGLTEARIQVWFQNRRAKWRKQEKVGPQGHPYNPYLSPGGGGPAPSVVAPSLPNPFNLPFGLRKPFDSLSFRYPPHVLPSYLPSPPGYHRGGPALLPPSVSLYPSASSFQTLLANISAAQRPKLPAQEFTTSPPLSPGGSAVTPPDVDRRSSSIASLRLKAREHELRLEMLRQNGDLIS

>gi|270011563|gb|EFA08011.1| hypothetical protein TcasGA2_TC005600 [Tribolium castaneum]

MFCYHCPPALHPGAPQPRLPTLEYPFTPTHPYTSYSYHPAIHDDTFVRRKQRRNRTTFTLQQLEELESAFAQTHYPDVFTREDLAMKINLTEARVQVWFQNRRAKWRKAERLKEEQRKRDGQDVVKRDQEETKEEKVEETSSPEVAGPDEELVRECSSRSSGRETPEQRVAETSPDPASPQSPAASASSEPPRPVPPPFSHMFPFGDSPQVWEAPASSKKLNLAGFPGLCSCCPIKPMAGLLPSGDGSQISPGGGDQRSSSVAELRRKAQEHSAALLQSLQHVANFQQAAGLASGLNFPLLPPLTLQALNRKHEMPETATNSKESTT

>gi|328792237|ref|XP_624630.3| PREDICTED: homeobox protein aristaless-like isoform 2 [Apis mellifera]

MGISTEEVRAEEESNHPRAAPTSPESETEVDDFAPKRKQRRYRTTFTSFQLEELEKAFSRTHYPDVFTREELAMKIGLTEARIQVWFQNRRAKWRKQEKVGPQAHPYNPYLGSSAPPASAVVAPTLPNPFAHLSTFALRKPFDAFRYPPLGHGPVLPGSYTAHPYHRAPPPLLPPGMPLPYTSAASFQSLLANISAAQRPKLVPPPPPQASSPQGSPTAATVGLPDIDRRTNSIASLRLKAREYELHLEMLRKNGDLIS

>gi|321473819|gb|EFX84785.1| hypothetical protein [Aristaless] DAPPUDRAFT_36325 [Daphnia pulex]

KRKQRRYRTTFTSYQLEELEKAFSRTHYPDVFTREELAMKIGLTEARIQVWFQNRRAKWRKQEK

>gi|524878803|ref|XP_005096083.1| PREDICTED: uncharacterized protein LOC101856173 [putative Al][Aplysia californica]
MGPAGRTNPGYFPEYFIKQKRAACSTKLEELEKAFAQTHYPDVFMREDLAMRINLTEARVQVWFQNRRAKWRKSERFAQQPGGGRPTGSVGEDGEGCGQEGDKSTEAINEHPHSPAAFPDRVPQTAHAHERRGDEMEAQTSAGGPGSFVEERKSEGRDEVLVEIKSERSQADQEGCGDDLERQGFEEAQARESDRDTEGQGRDDGEGEGEICVDKNEPDDCYEGSGPHGEQRRRCSQNEAIDYTARKTEESSSGTELAGEMTVDVEDEDDNNNTGDKRPLLPILNHHRHESHMMMNASPRRHEPPPYLGFPRHSPIRHEFMPPPAASDGGKLILNTPSSLEPLISPPYEGRKSLSPTSSTSPALSLTNTPSVAAGIPMTSDGRGGITSTAGASLSVDAPPPGFPVSPSLAFGHSGMTSYGNDPALGMLGLMPPLMWRSPDLALMAKMSRGALPFTQSLLAMNRPGLLSSMDGSRFKSGYDSLLSSRQFLSSHLPHPAFKGCLPFCMYCPPRSNGTGSNHIAGNCSGGGSNSGSFPPTFGEHRTSSLAELRQKAREHAEAIVARRTDSPTPPTD

>TSA: Schmidtea mediterranea smed020892 transcribed RNA sequence

Sequence ID: gb|GAKN01007720.1|Length: 432

RKQRRNRTTFTMQQLEELEKAFQQTHYPDVFTREELALRISLTEARVQVWFQNRRAKWRK

>gi|118343665|ref|NP_001071654.1| transcription factor protein [Ciona intestinalis]
MAQFSSYSIDRLLGHSGHKGSFILRHEDEGQPRVFAFSGSKSAQHHLERKFSQQESVLSKQDPPCYIPHDIHTLPSREQKQNGDRPFVPISAGEDNQQKLSAAKNSFNYQDHFLYLLRQQYDQAGIHTTTPIIANDEHRAAPGSERESTLNVRSKREDCVSDRRRSQSFTPPPISSPEQGSSPTSSNEHPCSSNEVSELTSSVQTSSPDDQDRNSSKRKQRRYRTTFSAFQLEELERAFQKTHYPDVFTREELAMRVDLTEARVQVWFQNRRAKWRKREKQGIFNSLGSIPGLSNVTSFPPIPMFMDPLLRHIYLSSSSKPDERQTMTQSSLQWRHLALAAAVQRMNCPTPTVPINFPRIGSVRSPLNFLANPVLSSNLSSQRPAHIYPHPYSSQRVPITAESKDTSTETDGNTPNRLHR
FATSSNDSI

>gi|260796221|ref|XP_002593103.1| Q50 paired-like homeodomain transcription factor [Branchiostoma floridae]

MTALKSPYSIEGILGRTSNPSRGSDGRGSVIVAGLGLIGPAPGAKESRSILVQKNCGGMNAEVATIGRAAGHEDNSGESAALDFSIKRKSAEDKKDLKQDLNRNHFSEKDIKKEDMHEGEDSGSESDCGPLGSDLPPCGSPTSSGAVHEDKLNDLCDSINGQVNSLAGIDLENGGKRKQRRYRTTFTSYQLEELERAFAKTHYPDVFTREELAMRVDLTEARVQVWFQNRRAKWRKREKAQAQQQAQMQLHHPGGLPGVHPYLDMGCAPHHPGEHPPGVPGLSCPPLSPLPPALHPGSFPLHPALGGGLLRTPLGPAGFSPFLRGAAPVFRPMLQVDGLQALAESDRRASSIATLRLKAKEHSAQIEALGQHAKPATLKEVCS

>2gi|6671541|ref|NP_031468.1| homeobox protein aristaless-like 4 [Mus musculus]

MNAETCVSYCESPAAAMDAYYSPVSQSREGSSPFRGFPGGDKFGTTFLSAGAKGQGFGDAKSRARYGAGQQDLAAPLESSSGARGSFNKFQPQPPTPQPPPAPPAPPAHLYLQRGACKTPPDGSLKLQEGSGGHNAALQVPCYAKESNLGEPELPPDSEPVGMDNSYLSVKETGAKGPQDRASAEIPSPLEKTDSESNKGKKRRNRTTFTSYQLEELEKVFQKTHYPDVYAREQLAMRTDLTEARVQVWFQNRRAKWRKRERFGQMQQVRTHFSTAYELPLLTRAENYAQIQNPSWIGNNGAASPVPACVVPCDPVPACMSPHAHPPGSGASSVSDFLSVSGAGSHVGQTHMGSLFGAAGISPGLNGYEMNGEPDRKTSSIAALRMKAKEHSAAISWAT

***Daphnia magna* r- opsin sequences:**

Dmag *UV r- opsin*

MLLPNVWPGLNNESTGPIAQSWKYESRMNGWNTPADYKSYVHPHWLSYEEPNPMLHHLLG VLYIFFMIASCLGNGIVIYIFSTTKELKTPSNILILNLAICDFIMMIKTPVFIVNSFNEG PVFGRLGCSIYGLLGAYVGPCSAVTNAAIAYDRYRCISDPMGKRWSKSQASLIVLGCWVY ASPVSLLPFTELVNRFVPEGYLTSCTFDYMADNLETKIFVFLLWIWCWIMPLGVIIFSYG KITTQVMTHEARLKEQAKKMNVETLRSGANKDVRNEIRVAKVGISLTTLFLLSWTPYFMI AFIGCYGNRALLTPGLSMIPACTCKLAACVDPFVYAINHPKYRLELMKRLPWLCVHEKDE CAKEESSNASVISEAESRT

Dmag *LW A-1 r- opsin*

MDNQLNASFAYRSAGKPPVVWGFPPGASIIDTVPEDMLDMIHPHWKKFPPVNPMWHYLLG LLYIVLGMASITGNSLVLHLFMKTKDLKTPANMFVVNLAFSDLCMMITQFPMFVLNCFNG GVWLFGPFMCELYACTGSVFGLCSICTMAAISYDRYNVIVNGMNGTRMTYGRAGAFILFC WAYAIGWSIPPFVGWGRYIPEGILDSCSFDYLTRDALTVSYTCSLFVSNYCTPLAIIIFC YYHIVGAIIHHEKALREQAKKMNVTSLRSNADQNAQSAEIRVAKVAMINISLWVAMWTPY AAIVLQGAIGNQENITPLVTILPALIAKSASIANPIIYAISHPKYRLALQTALPWFCINE KASPAGETQSQASACTTSTS

Dmag *LW A-2 r- opsin*

MSNLTDIQAFAYRAGEQMTWGYPPGASLVDTVPEDMRATIHPHWNKFPPVNPMWHYILGL SYIILGTISIFGNGLVLHLFSKTKELKTPANMFVVNLAFSDLCMMISQFPWFVYNSFNGG VWLFGPFLCELYSCTASVFGLGSITTMAAISYDRYNVIVNGMNGPRMTYKKAAGIITFCW CYAILWSIFPFLGWGAFIPEGILDSCGFDYLTRDIRIVSYTCCVFVSNYCTPLLIISYCY YHIVQAIFHHEKSLREQAKKMNVTSLRSNVDQNAQSAEIRIAKVALANIALWVGMWTPYA TIVLQGAVGNQDNITPMVTILPALICKCASIANPIVYAISHPKYRLALQKELPWFCINEK APTGTDTQSTGSAVTAASSDTAT

Dmag *LW A-3 r- opsin*

MENVMQEIMNPLSNANASNLIRTARAVQQNVYFGYPAGVSLTDFVTDDMKALVHPHWSKF PPVNPMWHYLLGTIYIFLGMISLFGNGVVISLFTKTKELRSTANMFVVNLAFSDFCMMVT QFPMFVHNSFNGGIWLFGPAACELYACTGSIFGATSICTMAVIAYDRYNVIVKGMSGTRM TSKKATILIACCWTYATVWSIMPYFGFGRYIPEGILDSCSFDYLTRDQQTKVFGLCLFFF LYCIPLSFITFCYYFIVKTIFEHENTLREQAKRMNVSSLRTNADSNATSAEIRIAKVALC NIALWAAMWTPYAAIVLQGLLGDQSSITPLVTILPALIAKSASICNPVVYAISHPKFRLA LQEKYPWFCINEPIKADNDSVCSTKTAVSSTPSEST

Dmag *LW B-1 r- opsin*

|  |
| --- |
| MSVRTFGSSTYPCLTGTAAVQFGTLAAVSKVNATKSDQQQKGQVQVFSIKQPIKSENVQH LFFGRKLRKHYYRMETNVTAMAQQQPFNPWAIPDSFTVYHFAPEEIRSFLHPHWQTQKAP HPMVYYLFGILYLVIGTVAVGGNYMVLRILGSFPSLRTPANMLVMNLAVSDFLLMITLLP EACYNFFLGGPWQFGDLGCQIHAFCGALCGYSQITTLVLISYDRYNVIVKGFNAAPLTFG KASAMIVFSWMYALGWSVCPLVGWGYYAMDGMLGTCSFDGLTTNMNNKSHILASTMFLYV IPIIVIVGCYYFIVKAVFHHEDELRQQAKKMNVTSLRSNSDQQAVSAEIRIAKVAIINVT LWIIAWTPFAVVCMLGTWGDTSKITPYICELPVILAKTSCAYNPVIYALSHPKYRECLKE LFPWLRQPVDWFD |

Dmag *LW B-2 r- opsin*

MAFSNSNVTAMAQQQPYNPWSLPDSFTIYNFAPEEIRSFLHPHWHNQKAPHPMMYYFFGL LYTVIGVVAISGNWMVLRILGSFPSLRTPANMLVMNLAVSDFLLMITLIPECVYNFFTGG PWQFGDLGCQIHAFCGALCGYSQITTLVFISYDRYNVIVKGFNAAPLTFSKAIMFITFGW VWALGWSVSPLVGWGYYAMDGMLGTCSFDGVTPDMNNKTHILSCFTVMFVIPVMIIICCY YFIVRAVFHHEDELRQQAKKMNVTSLRSNSDQQAVSAEIRIAKVAIINVTLWIMAWSPFA VVCMLGTWGDVSRITPLVCELPVILSKTSCAYNPIIYALSHPKYRECLKELYPWLCIVVE TKKPSKHGGDNQSAGSTKTEASATTQA

Dmag *LW B-4 r- opsin*

MNHSAEMTATALAQRTTFDPWSLPDTFTVFAFAPEDIRGFLHPHWHTQKSPHPMLYYFFG LYYLLMGSIAISGNIIALRIFGRNPALRTPANMLVMNLAMSDLLLMITLIPECVYNFFLG GPWQFGELGCQIHSFCGALFGYNQIMTLTIISWDRYNVIVKGFNGKPLTFSKSIILIIFS WIWALGWSIAPLLGWGLYAMDGMLGTCSFDAVTTTMNNKSYLMAAFVSNYVLTVSSIIIC YYFIVQTVFHHEDELRQQAKKMNVTSLRSNTDQQAVSAEIRIAKIAIMNVTLWLVSWTPF AVICLLGTWGDTSKITPLVSELPIILSKTSCAYNPIIYALSHPKYRECLKELYPWICIVP DSKGGKRGGETQSISSSKTQASEST
